# A precision neuroscience approach to estimating reliability of neural responses during emotion processing: Implications for task-fMRI

**DOI:** 10.1016/j.neuroimage.2023.120503

**Published:** 2023-12-22

**Authors:** John C. Flournoy, Nessa V. Bryce, Meg J. Dennison, Alexandra M. Rodman, Elizabeth A. McNeilly, Lucy A. Lurie, Debbie Bitran, Azure Reid-Russell, Constanza M. Vidal Bustamante, Tara Madhyastha, Katie A. McLaughlin

**Affiliations:** a Department of Psychology, Harvard University; b Phoenix Australia—Centre for Posttraumatic Mental Health, Department of Psychiatry, The University of Melbourne, Melbourne, VIC, Australia; c Department of Psychology, University of Oregon; d Department of Psychology and Neuroscience, University of North Carolina at Chapel Hill; e Rescale; f Department of Psychology, University of Pittsburgh; g Integrated Brain Imaging Center, University of Washington

**Keywords:** fMRI, Reliability, Neuroimaging, Longitudinal, Individual differences, Translational neuroscience, Clinical neuroscience

## Abstract

Recent work demonstrating low test-retest reliability of neural activation during fMRI tasks raises questions about the utility of task-based fMRI for the study of individual variation in brain function. Two possible sources of the instability in task-based BOLD signal over time are noise or measurement error in the instrument, and meaningful variation across time within-individuals in the construct itself—brain activation elicited during fMRI tasks. Examining the contribution of these two sources of test-retest unreliability in task-evoked brain activity has far-reaching implications for cognitive neuroscience. If test-retest reliability largely reflects measurement error, it suggests that task-based fMRI has little utility in the study of either inter- or intra-individual differences. On the other hand, if task-evoked BOLD signal varies meaningfully over time, it would suggest that this tool may yet be well suited to studying intraindividual variation. We parse these sources of variance in BOLD signal in response to emotional cues over time and within-individuals in a longitudinal sample with 10 monthly fMRI scans. Test-retest reliability was low, reflecting a lack of stability in between-person differences across scans. In contrast, within-person, within-session internal consistency of the BOLD signal was higher, and within-person fluctuations across sessions explained almost half the variance in voxel-level neural responses. Additionally, monthly fluctuations in neural response to emotional cues were associated with intraindividual variation in mood, sleep, and exposure to stressors. Rather than reflecting trait-like differences across people, neural responses to emotional cues may be more reflective of intraindividual variation over time. These patterns suggest that task-based fMRI may be able to contribute to the study of individual variation in brain function if more attention is given to within-individual variation approaches, psychometrics—beginning with improving reliability beyond the modest estimates observed here, and the validity of task fMRI beyond the suggestive associations reported here.

## 1. Introduction

Functional MRI (fMRI) was developed as a tool to investigate properties of brain function in humans. The classic approach to doing so involves contrasting the blood-oxygen-level-dependent (BOLD) signal while participants are engaged in a task designed to elicit a particular cognitive or affective state with BOLD signal during a relevant control condition (Huettel et al., 2004; Poldrack et al., 2011). This approach has stimulated substantial knowledge about the functional properties of specific brain regions (Garrison et al., 2013; Sergerie et al., 2008; Shenhav et al., 2013).

More recently, fMRI data have been used to examine differences in brain function across individuals. A recent meta-analysis and analysis of two large cohorts (Elliott et al., 2020) calls into question the validity of using task-based fMRI to study these types of between-person individual-differences in brain function, demonstrating low test-retest reliability for many common fMRI tasks. These findings suggest that the stability of task-related BOLD signal over time is relatively poor, which undermines the utility of fMRI as a brain-based biomarker (Elliott et al., 2020). Test-retest reliability was particularly poor for amygdala activation in tasks involving emotion processing, which have frequently been used to study individual differences in brain function. These types of tasks have been used frequently to investigate whether brain function varies as a function of mental health symptoms (Etkin and Wager, 2007; Groenewold et al., 2013), personality traits (Canli et al., 2001; Gray et al., 2005), or environmental experiences, such as early-life adversity (McLaughlin et al., 2019; Tottenham et al., 2011). Here, we investigate an alternative interpretation of this finding—that the BOLD signal measured in task-based fMRI is reliable, but exhibits high within-person variability over time.

The relatively low intra-class correlation coefficients (ICCs) observed in the recent meta-analysis (Elliott et al., 2020) mean that a participant who exhibits high activation in a particular region in response to a task at one time is not more likely to exhibit high activation in that same region when tested at a later time on the same task, relative to others in a sample. One possible source contributing to this variability in task-based BOLD signal over time is noise or measurement error in the instrument (Chen et al., 2018; Gonzalez-Castillo et al., 2017). In addition, the construct itself—brain activation elicited during fMRI tasks—may vary meaningfully across time within-individuals. In other words, variability in neural activation to tasks may be more state-like than trait-like. Indeed, influential recent work suggests that a wide range of psychological (e.g., affect) and physiological (e.g., heart rate) constructs exhibit greater variability within individuals than across individuals (Fisher et al., 2018). Variation in neural response to fMRI tasks may in part reflect high temporal variability (and so low test-retest reliability) in the construct itself, as well as measurement error. Examining the contribution of these two sources of test-retest unreliability in task-evoked brain activity has far-reaching implications for cognitive neuroscience. If test-retest reliability largely reflects measurement error, it suggests that task-based fMRI has little utility in the study of either inter- or intra-individual differences. On the other hand, if task-evoked BOLD signal as a construct varies meaningfully over time, it would suggest that this tool may still be well suited to studying intraindividual (i.e., within-individual) variation.

We use data from a unique longitudinal study involving monthly fMRI scans of an emotion processing task on the same individuals over the course of one year to characterize the degree of inter- and intra-individual variability in neural responses to affectively-salient cues and the reliability of these responses over time and the internal consistency of the BOLD signal within individuals. This intensive longitudinal approach aligns with the emerging field of precision neuroscience, which focuses on repeated sampling of fMRI data from the same individuals over time to examine patterns of stability and variability in neural function (Braga and Buckner, 2017; Gordon et al., 2017; Gratton et al., 2020; Laumann et al., 2015; Poldrack, 2017). Early precision neuroscience studies revealed dynamic fluctuations in brain function within individuals in networks previously thought to be stable based on between-participant designs (Poldrack et al., 2015). Influential recent work recommends within-participant longitudinal designs as a more powerful strategy for examining brain-behavior associations than cross-sectional brain-wide association studies (Marek et al., 2022). Neural responses during emotion processing may be particularly likely to vary within-individuals over time, given the high within-individual variation of affect (Rocke et al., 2009). Meta-analysis of these types of emotion processing tasks consistently reveal activation in amygdala as well as widespread cortical recruitment across regions in the salience network, such as anterior insula; the default network, including precuneus, posterior cingulate, medial prefrontal cortex, and middle temporal gryus; the frontoparietal network, including inferior, middle, and superior frontal gyrus; as well as the fusiform and other cortical regions in the ventral visual stream (Fusar-Poli et al., 2009; Sabatinelli et al., 2011). Despite these robust patterns at the group-level, the stability of neural responses in affective processing tasks over time was low in the recent meta-analysis (Elliott et al., 2020). To evaluate the degree to which this variability reflects meaningful within-individual fluctuations in neural responses versus measurement error, we estimate the internal consistency of the BOLD signal across the brain during an emotion processing task, within individuals, at each session. In doing so, we aim to contribute to the emerging debate about the reliability and utility of task-based fMRI for studying individual variation (Elliott et al., 2020; Kragel et al., 2021).

Characterizing the reliability of task-related BOLD signal is important because it puts an upper bound on our ability to detect valid associations with other measurements. In fact, an indirect indication of the reliability of a signal is the ability to detect expected associations with other constructs of interest. We are careful to note that there is not a one-to-one correspondence between reliability and the detection of associations. While higher reliability increases the ability to detect associations, larger effect sizes do the same; and while some reliability is necessary for detection, high reliability does not allow one to detect associations that are not present. However, detection of true associations suggests that reliability is high enough for a given effect size, with the important caveat that one does not know whether the detected effect is true or not. Moreover, as has been written about extensively elsewhere (Pashler and Harris, 2012), low reliability resulting in low statistical power increases the probability that any given result is a false positive, and with a publication filter on significant effects, inflates the proportion of false positives in the literature.

As such, we further investigate reliability by leveraging our dense sampling approach to determine whether we can predict variability in task-related BOLD response over time, within individuals. Specifically, we evaluate whether monthly fluctuations in mood, sleep quantity, and exposure to stressful life events (SLEs) are associated with changes in neural responses to affective cues over time, within individuals. These factors each fluctuate dynamically within individuals over time and have been associated with neural responses to aversive cues in between-person studies (Arnone et al., 2012; Goldstein-Piekarski et al., 2015; Larson and Ham, 1993; Mroczek and Almeida, 2004; Sliwinski et al., 2009; Swartz et al., 2015b, 2015a; Wang et al., 2006). These studies have demonstrated increases in activation of amygdala, anterior insula, and other regions of the salience network to aversive stimuli in individuals who have experienced high levels of SLEs (McLaughlin et al., 2019; Swartz et al., 2015b). Associations of sleep with neural responses during emotional processing are somewhat mixed and have largely focused on the amygdala. Greater sleep duration, particularly REM sleep, is associated with reduced amygdala reactivity to aversive stimuli (van der Helm et al., 2011; Wassing et al., 2019), whereas sleep deprivation predicts elevated amygdala reactivity (Yoo et al., 2007). A recent analysis from the UK Biobank observed the opposite pattern, however, with habitual short sleep associated with decreased amygdala reactivity (Schiel et al., 2022). Studies linking mood to neural responses to emotion processing have also observed that higher levels of negative affect are related to increased amygdala reactivity and decreased prefrontal recruitment to aversive stimuli (Bastiaansen et al., 2018; Forbes et al., 2011). However, influential recent work has shown that between-person associations often do not align well with within-individual associations of the same constructs for a range of psychological and physiological variables (Fisher et al., 2018). For this reason, we did not necessarily expect to find the neural regions that have been observed in between-person studies of mood, sleep, and SLEs to emerge in our within-person analyses. We are unaware of prior work examining whether fluctuations in mood, sleep, or SLEs are related to within-individual variation in neural responses to affective stimuli.

## 2. Method

Further information and requests for resources should be directed to and will be fulfilled by the lead contact, John C. Flournoy (john_flournoy@g.harvard.edu; jcflournoyphd@pm.me).

### 2.1. Data and code availability

Code and data repository: https://osf.io/zy92w/.

- De-identified human standardized fMRI data have been deposited at repositories listed above. They are publicly available as of the date of publication.
- All original code has been deposited at repositories listed above and is publicly available as of the date of publication.
- Any additional information required to reanalyze the data reported in this paper is available from the lead contact upon request. Please let the authors know immediately if any resources are missing from the public repositories so they can be updated.

### 2.2. Sample

The study was designed to examine within-individual variation in neural responses to affective stimuli and associations of that variability with other key constructs that fluctuate over time within-individuals. A sample of 30 female adolescents aged 15–17 participated in a year-long longitudinal study that included 12 in-lab assessments conducted each month (355 monthly assessments) with neuroimaging completed at 10 of the 12 monthly visits, excluding the baseline and final visit (292 scans). Given our within-individual approach, we aimed to limit between-person variability in sex and age, focusing on adolescent females given the high levels of interpersonal stressors and stress vulnerability in this group (Hankin et al., 1998; Lewinsohn et al., 1998; Rudolph and Hammen, 1999). Participants were recruited from schools, libraries, public transportation, and other public spaces in the general community in Seattle, WA between April 2016 and April 2018. Inclusion criteria included female sex, aged 15–17 years at study onset, possession of a smart phone with a data plan, and English fluency.

Participants were excluded based on the following criteria: IQ *<* 80, active substance dependence, psychosis, presence of pervasive developmental disorders (e.g., autism), MRI ineligibility (e.g. metal implants), psychotropic medication use, active safety concerns, and inability to commit to the year-long study procedure.

Twenty-two participants identified as White (73%), 4 as Asian (13%), 2 as Black (7%), and 2 as mixed race (7%). Participants’ income-to-needs ratios were computed based on their parents’ report of total combined household income and household size. Four participants were in families with income below the poverty line (i.e., income-to-needs ratio below 1; 13%), 12 participants between 1 and 3 (30%), and 13 participants between 3 and 10 (33%). One participant did not provide income information. All study procedures were approved by the Institutional Review Board at the University of Washington. Written informed consent was obtained from legal guardians and adolescents provided written assent. Participants were paid increasing amounts of money for each monthly visit, for a total of $905 in possible earnings (Table S1).

### 2.3. Emotional processing task

Participants completed an emotional processing task involving passive viewing of emotional faces, including fearful, happy, neutral, and scrambled faces. We focus on the contrast of fearful > neutral faces, to examine variation in neural responses to aversive stimuli. This is a widely used contrast in affective neuroscience thought to capture neural responses to the presence of a potential threat in the environment. Indeed, similar tasks assessing neural responses to aversive stimuli have frequently been used in studies aimed at identifying brain-based biomarkers associated with stress, psychopathology, and numerous other between-person characteristics (Arnone et al., 2012; McCrory et al., 2011; Monk et al., 2008; Swartz et al., 2015b, 2015a; Thomas et al., 2001; Tottenham et al., 2011).

The task was completed across one run that included twelve 18-second blocks (three blocks each of fearful, happy, neutral, and scrambled faces). Blocks were displayed in a pseudo-random order that ensured that no block type was displayed twice in a row. ITI blocks were inter-leaved between blocks of faces. During each block, 36 faces of different actors expressing the same emotion were displayed for 300 ms each, with a space of 200 ms following each face, based on prior face processing tasks (Somerville et al., 2004). The total duration of the task was 4.5 min. The task was intentionally designed to be brief, given evidence that the amygdala, hippocampus, and other temporal cortex regions involved in emotional processing habituate rapidly to emotional faces (Breiter et al., 1996; Fischer et al., 2003). Similar brief tasks have been used to capture neural responses to affective stimuli in large-scale data collection efforts, such as the Human Connectome Project and the UK Biobank (Barch et al., 2013; Miller et al., 2016; Somerville et al., 2018).

Participants were asked to respond to prompts unrelated to the faces with a button press during the task to ensure they were paying attention. Specifically, an image (e.g., a scene or object) was displayed at one point in the run. A second image was subsequently presented and participants had to indicate whether the image was the same or different. Otherwise, participants were only asked to keep their eyes open and view the faces. Faces were drawn from the NimStim stimulus set (Tottenham et al., 2009). The “calm” faces from this dataset were used as neutral expressions, as these expressions are potentially less emotionally evocative than neutral faces, which are perceived as negatively-valenced (Tottenham et al., 2013). The scrambled faces consisted of the images of neutral faces with the pixels scrambled so as to resemble random static. Task-related functional activation for this contrast, averaged across all participants and months, controlling for the effect of time (centered at the first month), is depicted in Fig. S1.

### 2.4. Image acquisition and pre-processing

Neuroimaging data were acquired using a Phillips Achieva 3T scanner using a 32-channel head coil at the University of Washington’s Integrated Brain Imaging Center. Anatomical scans (T1-weighted MPRAGE volumes; TR=2530ms, TE=3.5ms, flip angle=7°, FOV=256 × 256, 176 slices, in-plane voxel size=1mm3) were acquired for co-registration with functional magnetic resonance imaging (fMRI). Blood oxygenation level dependent (BOLD signal during functional runs was acquired using a gradient-echo T2*-weighted echo planar imaging (EPI) sequence. 37 3mm thick axial slices were acquired sequentially and parallel to the AC-PC line (TR=2s, TE=25ms, flip angle=79°, Inter-slice gap=.6mm, FOV=224 × 224 × 132.6, matrix size=76 × 74). Prior to each scan, four images were acquired and discarded to allow longitudinal magnetization to reach equilibrium.

Preprocessing was performed using FSL (Smith et al., 2004) and AFNI (Cox, 1996; Cox and Hyde, 1997), with the following steps applied to functional images: (a) motion and slice timing correction in FSL; (b) skull-stripping using AFNI’s 3dSkullStrip; (c) despiking using AFNI’s 3dDespike tool; and (d) for the purposes of whole-brain analysis, but not reliability and internal consistency analyses, smoothing with a 6mm full-width half-max kernel using SUSAN in FSL. Nuisance regressors entered into person-level models consisted of 6 rigid-body motion regressors as well as time-series extracted from white matter and ventricles entered to control for physiological noise (Behzadi et al., 2007). Outlier volumes in which framewise displacement exceeded 1mm, the derivative of variance in BOLD signal across the brain (DVARS) exceeded the upper fence, or signal intensity was more than 3 SD from the mean were excluded by regressing these volumes out of person-level models. First-level models were estimated on these preprocessed BOLD images. The resulting contrast images were registered first to a study-specific template (Ghosh et al., 2010; Huang et al., 2010; “Python Client - TemplateFlow,” n.d.), and then to the standard space of the Montreal Neurological Institute (MNI) template at 2mm resolution. Anatomical co-registration of the functional data from each monthly assessment with each participant’s T1-weighted image from that same monthly assessment and normalization were performed using Advanced Normalization Tools (ANTs). All transforms were concatenated so only a single interpolation would be performed.

### 2.5. Quantification and statistical analysis

#### 2.5.1. Reliability analyses

The term “reliability” can be used to refer to many kinds of consistencies between measurements, all of which have the shared goal of capturing how similar another measurement is expected to be using the same instrument (see Revelle and Condon, 2019 for a thorough review). Here, we focus on test-retest reliability and internal consistency as quantifications of two distinct kinds of consistency or reliability in the BOLD signal (though each is commonly used to index an instrument’s signal-to-noise ratio). We describe our approach to estimating each of these in detail below.

##### Anatomical Regions.

Analyzing parcellated brain data can aid complete reporting of effect sizes across the whole brain, and increase statistical power (Cosme et al., 2022; Flournoy et al., 2020). For this reason, reliability and internal consistency were assessed using the Schaefer 400 cortical parcellation scheme developed using both task-based and resting-state fMRI methods (Schaefer et al., 2018), as well as 14 anatomically-defined subcortical areas in the Harvard/Oxford subcortical atlas (brainstem; right and left accumbens, amygdala, hippocampus, caudate, pallidum, putamen, thalamus) and 24 size-matched control regions defined as spheres in right and left cerebral white matter and lateral ventricle from the Harvard/Oxford subcortical atlas. We included these control regions as a baseline comparison because we expect BOLD signal to have lower test-retest reliability and internal consistency in these regions than in cortical and subcortical regions. Each of these regions-of-interest (ROIs) were registered to each participant’s monthly T1 image using ANTs, as described above.

To create size-matched control ROIs in left and right white-matter and ventricles, we used a random subsample of 10 cortical parcels and all subcortical regions (*N* = 14) to define the size of ROI for size-matching. Our procedure was as follows.

1. Compute the volume, in voxels, of the target subcortical region or cortical parcel.
2. Randomly select a voxel coordinate from the control region (left or right white matter or ventricle).
3. Construct a sphere with a radius such that the volume of the sphere is equal to the volume of the target region or parcel.
4. Ensure that the sphere falls within the control region. If not, repeat steps 1–3.
5. Compute the relevant statistic using voxels within the defined sphere.

##### Test-Retest Reliability.

**Fig. 1.**
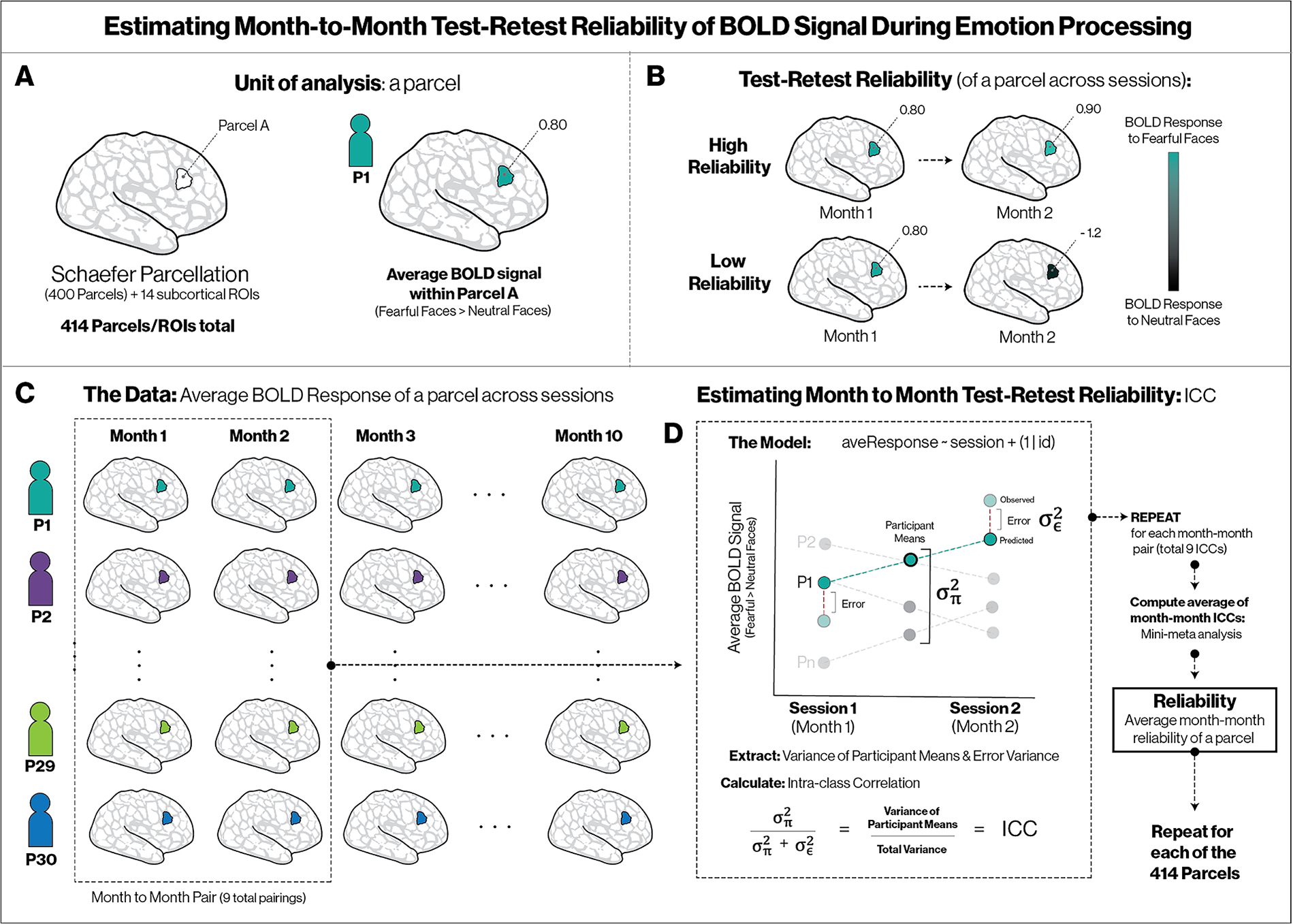
Methods used to estimate test-retest reliability. A: The unit of analysis is the mean of voxels in a cortical parcel or subcortical region of interest (ROI) for the contrast for Fear > Neutral measured for each participant at each month. B: Test-retest reliability (i.e., temporal stability) is operationalized as the similarity (in rank order of participants) of the BOLD signal in each parcel from month to month. C: The data are observations for a particular parcel from each participant, for each month; we estimate an ICC for each pair of adjacent months for that parcel. This yields N × S rows of data for each parcel, where N is the sample size and S is the number of sessions (months). D: To estimate test-retest reliability, we compute an ICC that decomposes the total variance into the variance in participant means across months and error variance, after conditioning on the group means for each session. The ICC is then computed as the variance due to participant means over the total variance. We do this for each pair of adjacent months and then compute an average ICC across all pairs using meta-analysis. We compute this overall ICC as the measure of test-retest reliability for each of the 414 parcels and subcortical ROIs, and 5 regions of no-interest (see Methods for details).

Test-retest reliability—a measure of temporal stability across participants in a sample (Chen et al., 2021, 2018; Koo and Li, 2016; Shrout and Fleiss, 1979)—was estimated by computing the ICC for BOLD signal in the Fear > Neutral contrast for each participant, at each session, in each cortical parcel and subcortical region (see Fig. 1). A Bayesian multilevel model (i.e., hierarchical linear model) was fit for each pair of adjacent sessions (e.g., sessions 1 and 2, sessions 7 and 8); estimating a single ICC across all 10 sessions for each participant produced consistently lower ICC estimates than this approach (see Fig. S2). Using *brms* (version 2.15.0; Bürkner, 2018, 2017) in R (v4.04; R Core Team, 2021) we fit the model

$$y_{ij}=\beta_{0}+\beta_{i}+\pi_{j}+\epsilon_{ij}$$

where $\beta_{0}$ is the overall mean (i.e., the grand mean across all participants), $\beta_{i}$ is the fixed effect of session i, and $\pi_{j}$ is the random intercept of participant j. The ICC was then calculated as

$$C_{pair}=\frac{\sigma_{\pi }^{2}}{\sigma_{\pi }^{2}\;+\;\sigma_{\epsilon }^{2}}$$

where $\sigma_{\pi }^{2}$ is the variance of π (i.e., random intercept variance) and $\sigma_{\varepsilon }^{2}$ is the residual (i.e., error) variance. ${ICC}_{\text{pair}}$ corresponds to the ICC (3,1), also referred to as consistent agreement, (Chen et al., 2021, 2018; Koo and Li, 2016; Shrout and Fleiss, 1979) and reflects the proportion of variance due to participant means across sessions, and is also the expected correlation between observations across sessions from the same participant (Chen et al., 2018). Bayesian estimation was chosen primarily because it allows straightforward computation of credible intervals of the quantities of interest. The resulting posterior distributions were logit-transformed to rescale them from [0,1] to (-∞,∞) and the medians and standard deviations were meta-analyzed using brms to obtain a single estimate of individual consistency between temporally adjacent sessions. The meta-analytic model was

$${ICC}_{pair}\sim Normal\left({ICC}_{\text{true}},{ICC}_{SE}\right)$$

$${ICC}_{true}\sim Normal(\mu ,\sigma )$$

where ${ICC}_{pair}$ is the set of observed pairwise ICC estimates, ICC true is the latent true ICC, ${ICC}_{SE}$ is the set of uncertainties (standard deviations) in the observed ${ICC}_{pair}$ estimates, and μ and σ are the mean and standard deviation, respectively, of ${ICC}_{true}$. The resulting posterior of parameter μ was logit^−1^-transformed to rescale back to [0,1] and the resulting median and 95% credible interval were interpreted as estimates of test-retest reliability of neural responses for each region across all individuals in the sample.

This approach to estimating reliability evaluates the stability of neural responses in each region within participants over time by evaluating how much the data from each participant deviates from their person-level mean across sessions. Higher estimates reflect higher test-retest reliability in BOLD signal across participants (i.e., a high ICC implies that a participant with high BOLD signal in a particular region in a particular session, relative to others in the sample, is also likely to have high BOLD signal in that region in other sessions).

##### Internal Consistency.

**Fig. 2.**
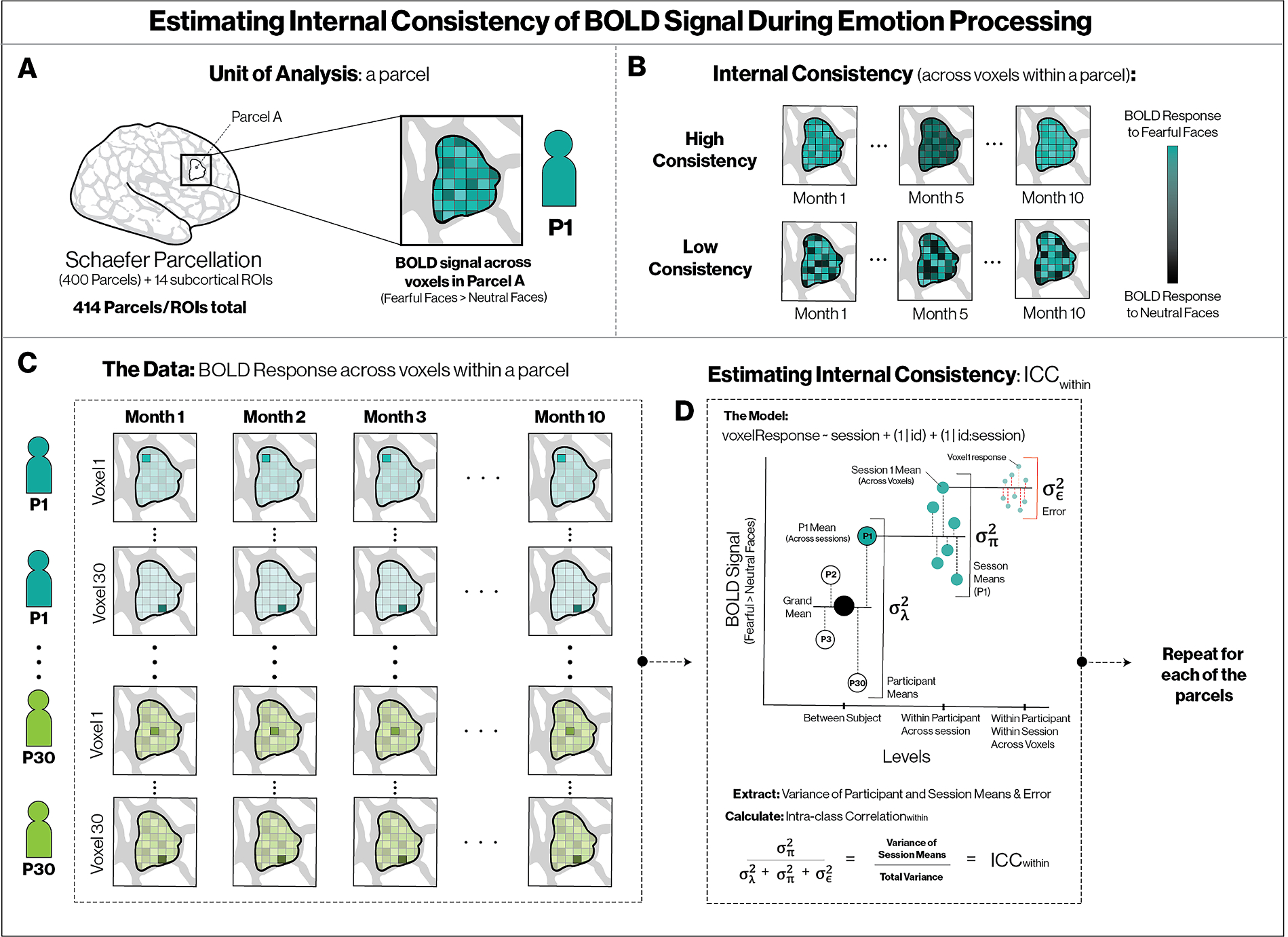
Methods used to estimate internal consistency. A: The unit of analysis is the voxel contrast parameter value within a cortical parcel or subcortical region of interest (ROI) from the contrast for Fear > Neutral measured for each participant at each month. B: Internal consistency is operationalized as the similarity (in rank order of voxels) of the signal across participant-sessions; high internal consistency means that voxels within a parcel for one participant-session tend to be similar to one another as compared to variation across participant-sessions. C: The data are observations for a particular voxel within a parcel from each participant, for each month; we estimate an ICC based on all voxels in that parcel. This yields N × S × V rows of data for each parcel, where N is the sample size, S is the number of sessions (months), and V is the number of voxels in the parcel. D: To estimate internal consistency, we compute an ICC that decomposes variance into the variance in participant means across all months, the mean for each session for each participant, and error variance, after conditioning on the group means for each session. The ICC is computed as the variance in participant-session means over the total variance. This is computed for each of the 414 parcels and subcortical ROIs, and 5 regions of no-interest (see Methods for details).

Low test-retest reliability is partly a function of the sources of variance that contribute to the magnitude of the error variance term, $\sigma_{\varepsilon }^{2}$, in the denominator. This term captures all unmodeled variance, and can reflect a mixture of high measurement error in measuring BOLD signal and high within-individual variability in responses over time. The internal consistency of multiple indicators of the same construct, based on generalizability theory (Bonito et al., 2012), is an alternative to the test-retest approach to quantifying instrument consistency that can disaggregate these sources of error. Reliable indicators that exhibit high within-individual variability (i.e., that fluctuate together over time), would produce high internal consistency, but low test-retest correlations. In these data, we can disaggregate variance not only into temporal stability across participants (reflecting test-retest correlations), but also into consistency across voxels at each session (reflecting internal consistency). This is one way to begin to approach quantifying the extent to which low test-retest reliability is a result of measurement error versus fluctuations of an internally consistent signal over time (Fig. 2). This approach is similar to evaluating the internal consistency of items on a scale by computing Cronbach’s alpha, which is based on the average inter-item correlations (Fig. S3). Here, instead of items on a scale we evaluate the stability of BOLD signal across voxels within distinct anatomical regions. As we would do with items on a self-report scale, we use voxel responses within person, within session, to estimate the reliability of the measurement instrument (in this case, parcels and subcortical regions). If a scale has good internal consistency, when the value on a single item increases for a participant at a particular administration, the values of other items on the scale should similarly increase. Equivalently, if the BOLD signal is measured consistently across voxels within individual, within session, when the contrast value in a voxel increases for a participant in a particular session, the value of other voxels in that parcel or subcortical region should similarly increase (Fig. S3).

Internal consistency was estimated for the same set of parcels and subcortical regions described above to determine the proportion of variance accounted for by consistency in BOLD signal across the voxels within each region for each participant at each session. This approach examines the degree to which BOLD signal for the voxels in a single parcel or subcortical region fluctuate consistently with one another within each session for each participant (i.e., participant-sessions). To do so, we fit a multilevel model using *brms* to data from each parcel across all sessions with the form

$$y_{ijk}=\beta_{0}+\beta_{i}+\lambda_{j}+\pi_{jk}+\epsilon_{ijk},$$

where $\beta_{0}$ is the overall mean, $\beta_{i}$ is the fixed effect of session $i,\lambda_{j}$ is the random intercept of participant j, and $\pi_{jk}$ is the random intercept for each participant j ‘s session k. From this we get estimates of the variance in each participant’s mean across sessions $\left(\sigma_{\lambda }^{2}\right)$ the variance due to means of voxels (as deviations from participant means) within each participant-session $\left(\sigma_{\pi }^{2}\right)$, and error variance $\left(\sigma_{\varepsilon }^{2}\right)$—the variance in deviations of each voxel value from the value implied by participant means plus participant-session means. The proportion of variance due to the mean of each participant-session reflects internal consistency of voxels within a parcel, which we calculate as

$${ICC}_{within}=\frac{\sigma_{\pi }^{2}}{\sigma_{\lambda }^{2}\;+\;\sigma_{\pi }^{2}\;+\;\sigma_{\epsilon }^{2}}.$$

The medians and 95% credible intervals of the resulting posterior distributions of ${ICC}_{within}$ were interpreted as the voxel-to-voxel consistency of within-individual, within-session neural responses in each parcel.

This approach estimates how much the data from voxels within a specific region deviate from the mean from all voxels in that region, for that participant in that session; this estimate is conceptually similar to the internal consistency of items in a scale commonly used as an estimate of the reliability of self-report scales. Higher estimates reflect higher consistency in BOLD signal across voxels in a parcel for each participant, at each session (i.e., the BOLD signal varies in a consistent way for all voxels in a particular parcel or subcortical region for each participant, at each session). Note that unlike the above ${ICC}_{within}$ equation, the equation for multilevel internal consistency also divides the error variance term by the number of items. Given that the number of voxels in each of our parcels is large (median number of voxels per parcel = 301, IQR 221 – 389), we report the raw proportion of variance instead, which is a more conservative approach. Also note that this metric was evaluated using the unsmoothed data. Smoothing is a standard pre-processing step in task-based fMRI analysis, but will inflate estimates of the true variance ratio within a parcel because it eliminates some amount of the unshared (i.e., error) variance. In order to further ameliorate the potential influence of smoothness we additionally computed ICCs for random subsets of 15 voxels from each parcel rather than from every voxel within a parcel.

Other approaches for estimating reliability of BOLD signal in task fMRI have been developed for event-related designs using individual trials as the unit of measurement (Chen et al., 2021). These methods cannot be applied here given the block design of our task and the strong habituation that occurs in both cortical and subcortical regions during emotion processing tasks (Breiter et al., 1996; Fischer et al., 2003).

##### Smoothness and Internal Consistency.

Given that other properties of the BOLD signal aside from task-related neural responses may contribute to correlations of voxels that are spatially proximal, we consider these estimates as an upper bound of the amount of variance in BOLD signal over time that could reflect internally consistent within-individual variation. To probe whether these other properties of the BOLD signal are driving internal consistency estimates, we additionally assess the association between smoothness of the spatial signal and our measure of internal consistency. For each cortical parcel, subcortical ROI, and size-matched control region, for each participant, for each session, we estimated the smoothness of the first-level residuals using 3dFWHMx in AFNI (Cox, 1996; Cox and Hyde, 1997). We then regressed the estimates of internal consistency on the number of voxels in each parcel and the average smoothness of each parcel, each allowed to vary as a function of anatomy classified as cortex, subcortex, and control region. Each predictor was encoded using smooth functions (Wood, 2017), and the internal consistency outcome was modeled as distributed beta to account for the bounded values in [0, 1]. We compared this model to a constrained model including only the linear effect of parcel size and random intercept by anatomy (see supplemental text for more details). Model comparison employed efficient approximate leave-one-out cross--validation (Vehtari et al., 2018), which provides an expected log pointwise predictive density difference (ΔELPD) between models as well as standard errors of that difference. Models were considered non-equivalent if the absolute size of the ΔELPD exceeded two standard errors, and the simplest (i.e., constrained) model was retained in absence of evidence of non-equivalence.

#### 2.5.2. Predictors of longitudinal within-individual variation in brain activity

##### Mood.

Negative affect was assessed using the Positive and Negative Affect Scale (PANAS), a twenty-item measure assessing positive and negative affect (Watson et al., 1988). The general form of the PANAS was used, and participants were asked to indicate the extent to they felt this way over the past month. Participants respond to each affective state (e.g., excited, interested, nervous, hostile) on a 5-point Likert scale ranging from 1 (very slightly or not at all) to 5 (extremely). The PANAS has excellent internal consistency and has demonstrated convergent, discriminant, and predictive validity in a number of investigations (Watson et al., 1988; Watson and Walker, 1996). The PANAS was administered at each monthly visit to assess mood over the month since the previous visit.

##### Sleep.

Daily sleep duration was assessed via an actigraphy wristband participants wore continuously for the duration of the study. The wristbands used accelerometer data collected in 1-min epochs and proprietary algorithms to detect sleep and awake states. These devices have been validated against polysomnography and EEG, with excellent sensitivity (i.e., ability to detect true sleep), and adequate specificity (i. e., ability to detect true wake; de Zambotti et al., 2016; Liang et al., 2018). We collected a total of 6824 daily sleep observations. We computed daily sleep duration in hours, aggregating over potentially multiple sleep events in the same day (e.g., naps, fragmented night sleep), and not including awake time between sleep events. Daily sleep duration was computed in hours each day for the 24-h periods from 7pm to 7pm, and then averaged across the two weeks occurring prior to each scan session. See supplement and Vidal Bustamante and colleagues (2020) for more information on the actigraphy devices and missing data.

##### Stressful Life Events (SLEs).

SLEs occurring in the past month were assessed at each visit using the UCLA Life Stress Interview, a semi-structured interview designed to objectively measure the impact of life events (Hammen, 1988). The interview assesses acute life events/episodic stressors (e.g., failing a test, break-up of a romantic relationship) and chronic stress (e.g., ongoing conflict in the home, long-term medical issues). The interview has been extensively validated and adapted for use in adolescents (Daley et al., 1997; Dohrenwend, 2006; Dohrenwend and Shrout, 1985, 1985; Hammen, 1991). Structured prompts are used to query numerous domains of life (i.e., peers, parents, household/extended family, neighborhood, school, academic, health, finance, and discrimination). Each reported stressor is probed to determine timing, duration, severity, and coping resources available. Research personnel objectively coded the severity of each experience on a 9-point scale ranging from 1 (none) to 5 (extremely severe), including half-points. Following prior work, a total episodic stress score was computed by taking the sum of the severity scores of all reported events, which reflects both the number and severity of acute stressors (Hammen et al., 2000), hereafter referred to SLEs. If the participant did not report any SLEs, they received a score of zero for that month. The severity of chronic stressors occurring in each domain were coded on the same scale. The chronic stress score for the domain where the participant was experiencing the highest amount of ongoing stress was used in analysis. The interview was administered at each monthly visit to assess SLEs and chronic stress occurring since the previous visit.

#### 2.5.3. Modeling longitudinal within-individual variation in brain activity

To evaluate whether mood, sleep, and SLEs were associated with within-individual variation in neural response to aversive cues, we implemented voxel-wise and parcel-level multilevel models designed to disaggregate between- and within-person variation in longitudinal data. A frequentist power analysis from simulation indicates that we have 80% power to detect a standardized regression coefficient of .17 (see supplement for power across effect sizes and further details).

##### Neuropointillist.

Traditional software packages for analyzing task-based fMRI data are limited in the types of statistical models that can be estimated to examine predictors of task-related activation and involve a number of meaningful limitations when examining longitudinal data—for a lengthy discussion of this issue, see Madhyastha and colleagues (2018). As a result, most longitudinal fMRI studies using complex longitudinal modeling approaches have extracted BOLD signal from ROIs and estimated models outside of fMRI software packages (Braams et al., 2015; Ordaz et al., 2013), limiting the analysis to specific ROIs rather than taking a whole-brain voxel-wise approach, which remains a common approach to analyzing fMRI data in cognitive neuroscience.

To address this issue, one of the authors of this work (TM) developed an R package ‘Neuropointillist’ that allows any model that can be specified in R to be estimated in fMRI data in each voxel of the brain (Madhyastha et al., 2018). Neuropointillist assembles longitudinal pre-processed and spatially normalized longitudinal fMRI data into a long-form dataset, where each row represents data from a particular voxel in a particular participant at a particular time. Neuropointillist accepts a model to be executed on each voxel in the dataset, written as a function called from R. The specified model is applied to every voxel, for every participant, and each measurement occasion. This affords complete flexibility to evaluate any statistical model of interest for voxel-wise fMRI analysis, including longitudinal models. The statistical parameter estimates obtained from first-level analyses can also be imported into traditional fMRI packages. See Fig. S4 for greater detail. Neuropointillist is freely available; for details and documentation see: http://github.com/IBIC/neuropointillist.

##### Person-level models.

Person-level models were estimated in FSL. Task-related regressors were created by convolving a boxcar function of phase duration with the standard (double-gamma) hemodynamic response function for each condition of the task (fear, happy, neutral, scrambled). A general linear model was constructed for each participant.

##### Longitudinal Analysis, voxel-level.

Individual-level estimates of BOLD activity were submitted to group-level random effects models using Neuropointillist (Madhyastha et al., 2018). Voxel-wise models were implemented in R (R Core Team, 2021) using the *nlme* package (Pinheiro et al., 2020) using restricted maximum likelihood (REML), with the intercept allowed to vary randomly across participants. This method is robust to bias when data are missing at random (Matta et al., 2018). We did not employ Bayesian estimation in this case due to its high computational cost. We first estimated unconditional models including a term only for time to examine linear changes in voxel-wise BOLD signal across the ten sessions. To dissociate between- and within-person effects of SLEs, we used within-individual centering (i.e., centering each participant’s monthly observations around their person-specific mean across the year-long study period) and between-participant centering at the year-level (i.e., centering each participant’s mean for the entire study period relative to the overall mean for the entire sample). This approach orthogonalizes variation in a given predictor into between- and within-person components (Enders and Tofighi, 2007), accounting for the dependent nature of the data both over time and within-participant, while controlling for trait-level characteristics of the predictor (i.e., average level of negative affect, sleep duration, or severity of SLEs across the entire year). We estimated five models predicting BOLD signal for the contrast of fearful > neutral faces using the following predictor, each decomposed into between- and within-individual components as described above: 1) negative affect; 2) positive affect; 3) sleep duration; 4) acute SLEs; and 5) chronic stress. This approach allowed us to examine variation in BOLD signal as a function of within-individual variation in each of these factors after controlling for average between-person differences in each.

Voxel-wise models included a main effect of time (coded as the number of months since the first study visit), and these within-person and between-person centered stress variables as fixed effects. We used the *clubSandwich* package (Pustejovsky, 2019) to compute cluster-robust standard errors in the presence of possible autocorrelation (Pustejovsky and Tipton, 2018), and apply the Satterthwaite correction to the degrees of freedom used to compute the *p*-value of the coefficient test. This *p*-value was converted to a *Z*-score and used as the test-statistic.

To correct for multiple comparisons in whole-brain analyses, family-wise error (FWE) rate was controlled at *α*=.05 for each model using Equitable Thresholding and Clustering (ETAC) cluster correction implemented in AFNI (Cox, 2019). The ETAC method allows detection of both small and large clusters by establishing multiple combined cluster-forming p-value/cluster-size thresholds that together control the FWE across permuted brain maps. For each model, 1000 permutations were generated. The resulting 1000 permuted z-score maps were then used to generate (using *3dXClustSim*) and apply (using *3dMultithresh*) the ETAC thresholds to the statistical parameter maps from the group-level analysis.

We generated 1000 permutations for each image using the following procedure. Specifically, first, permutations matrices were generated appropriately for nested data by shuffling observation indexes within participants (Winkler et al., 2015, 2014). We then implemented the procedure described by Freedman and Lane (Freedman and Lane, 1983) as follows:

1. regress the dependent variable (Y) on covariates (i.e., time, and group-centered mean scores of the dependent variable), saving the residuals and the predicted values of Y;
2. permute the residuals according to the ith row of the permutation matrix, then add the permuted residuals to the predicted Y values to produce Y*;
3. regress Y* on the within-person centered variable of interest, X, and covariates, and save the permutation test statistic for the association between X and Y*.

As in the group-level model, we used cluster-corrected standard errors to derive a *t*-statistic with Saterthwaite-approximated degrees of freedom. The *p*-value of this *t*-statistic was transformed to a *Z*-score, and saved as the permutation test statistic. The resulting 1000 permuted *Z*-score maps were then used to generate (using *3dXClustSim*) and apply (using *3dMultithresh*) the ETAC thresholds to the statistical parameter maps from the group-level analysis.

Significant clusters reveal regions of the brain where BOLD signal systematically increased or decreased during months when participants had greater negative affect, positive affect, sleep duration, or exposure to stress than was typical for them across the year.

##### Longitudinal Analysis, parcel-level.

For each parcel in the Schaefer 400 cortical parcellation scheme (Schaefer et al., 2018), as well as the 18 anatomically-defined subcortical areas in the Harvard/Oxford, we extracted voxel-level estimates for each participant-session. We then estimated the same model as above using bayesian estimation to be consistent with the reliability and internal consistency analyses at the parcel level in R (R Core Team, 2021) with *brms* (version 2.15.0; Bürkner, 2018, 2017), which averages across the voxels in a parcel in a model-based way. We obtained a posterior probability distribution for the estimate of each parameter of interest. Using this posterior distribution, we threshold the image so that the combined probability of making an error in the sign (Gelman and Carlin, 2014) of any of the coefficients is constrained to be less than 5%. To implement this, we first compute the proportion of the posterior distribution that has the same sign as the median, ordering the parcels from the largest to smallest value. We then compute the cumulative product, which is the probability of not making a sign error. We decide to interpret and display the set of coefficients that are most likely in the correct direction and that maintain the probability of sign error at *<* 5%. We also present uncorrected analyses examining effects in left and right amygdala for each predictor because so much previous work has focused on this region. These results are presented in the supplement.

## 3. Results

### 3.1. Test-retest reliability

We first estimated ICCs across adjacent sessions to examine test-retest reliability (i.e., temporal stability) of neural activation in response to aversive cues (fearful > neutral faces) within 400 cortical parcels, 14 subcortical regions, and 4 control regions (see Fig. 1 and Methods for details). Higher ICCs reflect higher test-retest reliability in BOLD signal across participants (i.e., a participant with high BOLD signal in a particular region in a particular session is also likely to have high BOLD signal in that parcel on other sessions, relative to others in the sample).

**Fig. 3.**
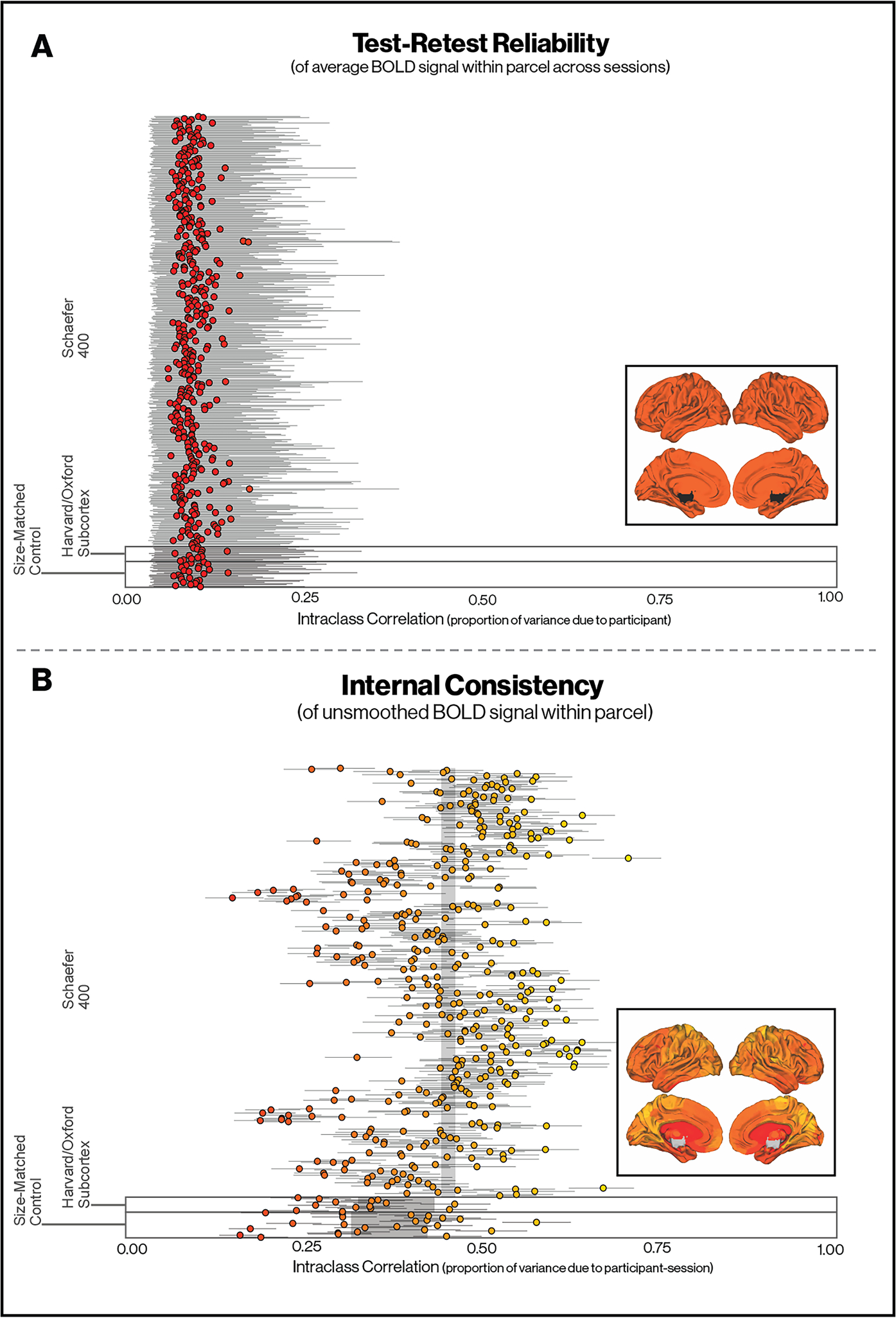
Test-Retest Reliability and Internal Consistency of fMRI response to aversive stimuli. A: Proportion variance due to participant means (test-retest reliability) across 400 cortical parcels,14 subcortical ROIs, and 24 size-matched control regions. Point estimates are filled with a color corresponding to the ICC, which maps onto the parcellated surface (bottom right corner) with whiskers showing 95% credible intervals. B: Proportion variance due to participant-session means (internal consistency), annotated as in (A). Shaded region indicates 95% confidence interval of expected value from the best fitting model predicting internal consistency for each anatomy type (see methods).

ICCs for test-retest reliability were uniformly small in magnitude and close to zero: ranges and interquartile intervals (IQR) for median posterior ICCs were ICC = [.04, .15] (IQR .06-.08; *N* = 400) across parcels, ICC = [.05, .12] (IQR .07-.08; N = 14) across sub-cortical regions, and ICC = [.04, .12] (IQR .06-.08; *N* = 24) across size-matched control regions (see Fig. 3A). This pattern indicates that the test-retest reliability of BOLD signal in response to aversive stimuli (fearful > neutral faces) is uniformly low across the brain.

### 3.2. Internal consistency

Next, we estimated the internal consistency of parcels and subcortical regions across voxels (see Fig. 2 and Methods for details). We used the same cortical parcels, subcortical regions, and control regions used to compute test-retest reliability to evaluate the internal consistency of the signal. Higher ICCs reflect greater consistency in BOLD signal across voxels in a particular region for each participant, at each session (i.e., the BOLD signal varies in a consistent way for all voxels in a particular region for each participant, at each session). The degree of internal consistency would indicate whether signal changes across these cortical parcels and subcortical regions reflect coherent signal rather than random fluctuations, and so inform the degree to which measurement error and high within-individual variation contributes to poor test-retest reliability in neural responses to aversive cues (fearful > neutral faces).

ICCs were substantially higher in magnitude than for test-retest reliability: ranges and IQRs for median posterior ICCs were ICC = [.13, .70] (IQR .37-.51) across cortical parcels, ICC = [.22, .45] (IQR .26-.34) across sub-cortical regions of interest, and ICC = [.14, .57] (IQR .28-.41; *N* = 24) in size-matched control regions (see Fig. 3B). These patterns demonstrate substantially higher within-person, within-session internal consistency than test-retest reliability. Estimates in subsamples of 15 voxels within each cortical parcel or subcortical ROI correlated nearly perfectly (*r* = .96) with estimates from all voxels. We interpret these patterns to suggest that the low test-retest reliability across sessions reflects, in part, internally consistent intraindividual variability in neural responses to aversive cues over time.

As a point of reference, we additionally compute Cronbach’s *alpha* as another commonly used metric of internal consistency. The primary difference between the ICC and and *alpha* is that *alpha* scales the error variance by the inverse of the number of items. As would be expected, this increases the estimate when using all voxels, *α* = [0.98, 1.00] (IQR 0.99–1.00), or even when a subset of 15 voxels are selected randomly from parcels (in order to keep scaling factor of the error variance reasonably small), with *α* = [0.65, 0.97] (IQR 0.90–0.94) for cortical parcels, and *α* = [0.81, 0.93] (IQR 0.84–0.88) for subcortical regions.

### 3.3. Smoothness and internal consistency

**Fig. 4.**
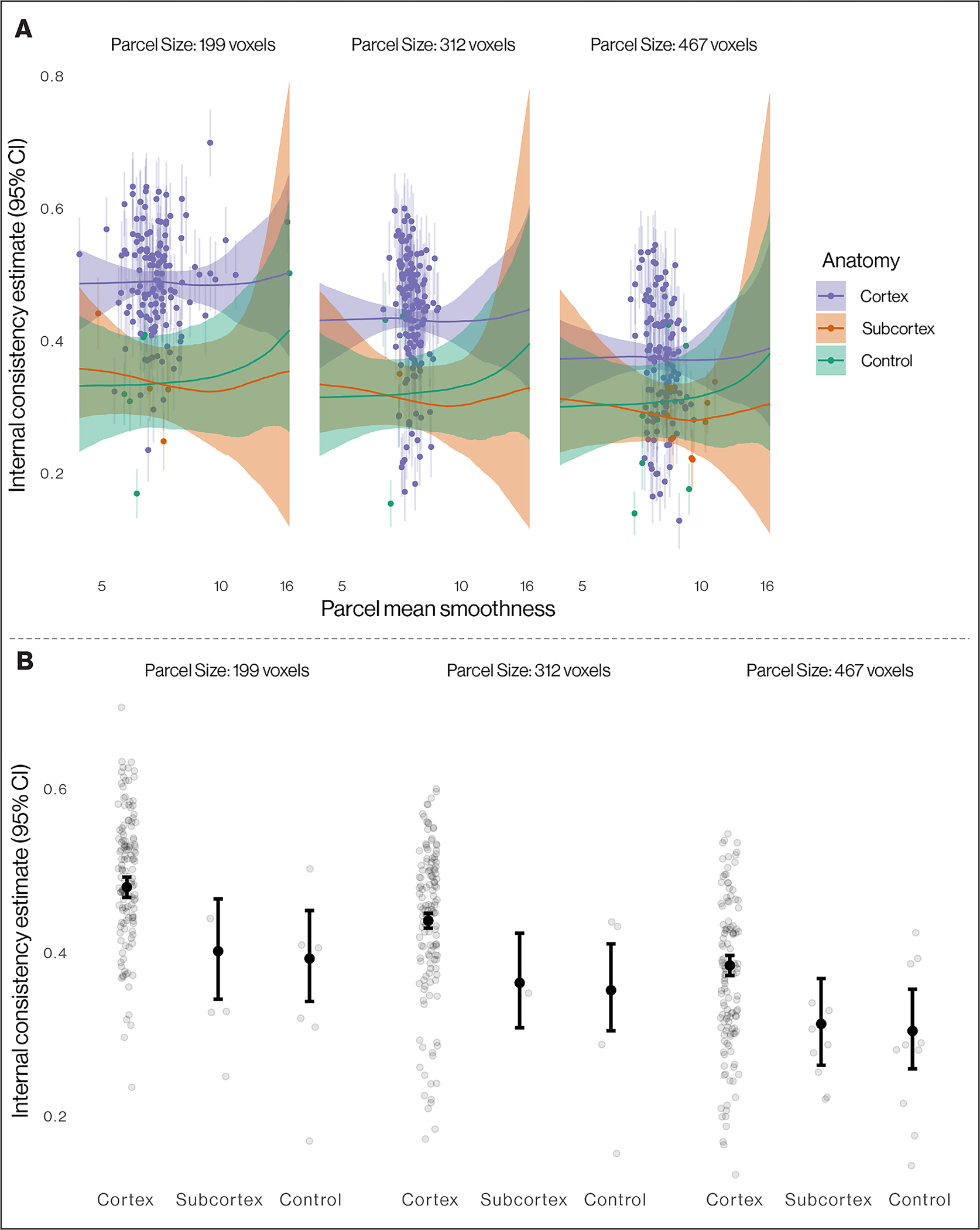
Effect of smoothness, size, and anatomy on internal consistency. Each panel shows model-expectations when the parcel size is as indicated in the panel titles, corresponding to the 1/6, 3/6, and 5/6 quantiles. Overlaid data come from parcels with sizes in a range centered on those quantiles (i.e., within the first, second, and third third of the data). A: Association between smoothness and internal consistency in the fully unconstrained model. The x-axis has been rescaled to foreground regions with the highest density of data. Whiskers on data points show the 95% credible interval for internal consistency estimates. B: Model expectations from the best fitting model highlighting expected differences in internal consistency between anatomy types. Cortex: Schaefer 400 parcels; subcortex: Harvard-Oxford subcortical regions; control: size-matched control regions.

Given the spatial nature of the analysis, to determine how smoothness contributes to the internal consistency observed we conducted additional analyses to examine the association of smoothness with estimates of internal consistency. A model including smoothness did not fit significantly better than a model with only parcel size and anatomy, and in fact fit worse (ΔELPD = −31.2, SE = 23.9; see Methods for a description of the ELPD), indicating that BOLD signal smoothness was not associated with internal consistency estimates after controlling for parcel size; this is also visible as nearly flat trends in the effect of smoothness in the plots (Fig. 4A; see full model output in supplement, and Fig. S5 for zero-order association of all three predictor variables). The model-expected internal consistency (from the constrained model) for each anatomy type at the median parcel size (312 voxels) were: cortex, .44 (95% CI = [.43, .45]); subcortex, .36 (95% CI = [.31, .42]); and size-matched control, .35 (95% CI = [.30, .41]; Fig. 4B), suggesting greater internal consistency in cortical parcels.

### 3.4. Changes in neural response to aversive cues over time

**Fig. 5.**
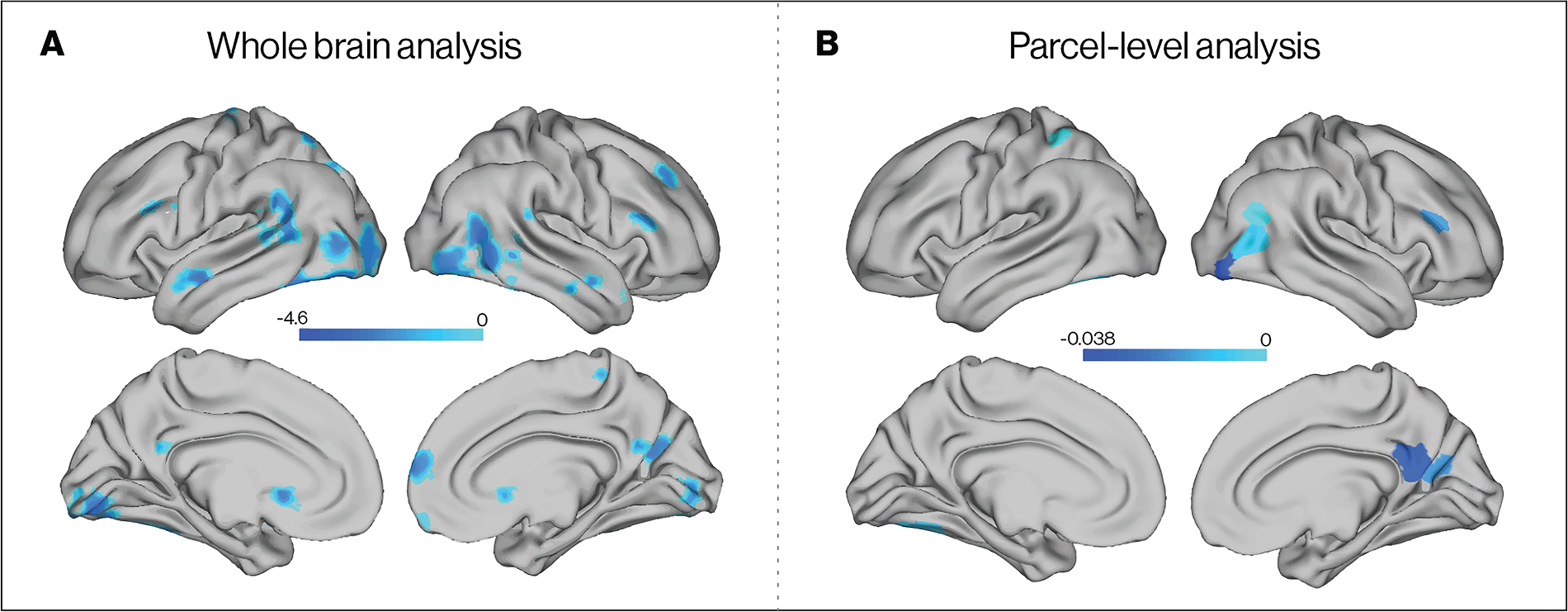
Effect of time for contrast of Fear > Neutral. A: Statistical map represents t-scores for the effect of study month on the contrast between viewing Fear faces versus Neutral faces; values are plotted for voxels within clusters determined to be significant. B: Parcels with 99.988% credible intervals excluding 0 are displayed using colors representing the effect size for each parcel in terms of standard deviations of the parcel-level outcome for a unit change in the predictor on its original scale.

Before examining correlates of within-individual variation in neural response to aversive cues (fearful > neutral faces), we first estimated unconditional growth models across the ten monthly scans. Linear decreases in neural response in this contrast were observed in the ventral visual stream, including fusiform and lateral occipital cortex; superior temporal sulcus; dorsomedial prefrontal cortex (PFC); and right middle frontal gyrus (MFG) and inferior frontal gyrus (IFG) (Fig. 5; Tables S2–S3). This pattern of habituation across monthly sessions is broadly consistent with evidence in the literature for within-session habituation to emotional faces in medial and lateral temporal cortex (Fischer et al., 2003). There were no regions where linear increases in activation over time occurred.

### 3.5. Predicting within-individual variation in task-related neural activation

Having demonstrated some internal consistency of neural responses to aversive cues, we next attempted to explain the fluctuations in these responses within-individuals over time. Specifically, we examined whether within-individual variation in mood, sleep, and stressful life events across time were associated with variability in neural response to aversive stimuli (i.e., fearful > neutral faces) in voxel-wise multilevel models that partitioned variance in stress into within-individual and between-individual components (see Methods for details). These variables were not highly correlated within-person (*ρ* = [−.11, .25]; see Table S4 for the full between- and within-person correlation table), so we did not attempt to isolate the unique effect of each variable while controlling for the others. Clusters reflect regions of the brain where monthly fluctuation in the predictor (i.e., mood, sleep, or stress) is associated with corresponding within-individual change in neural response to aversive cues (fearful > neutral faces). Across all analyses, parcel-level analyses seemed more sensitive with more widespread associations than in whole-brain analysis with cluster correction.

#### Mood.

**Fig. 6.**
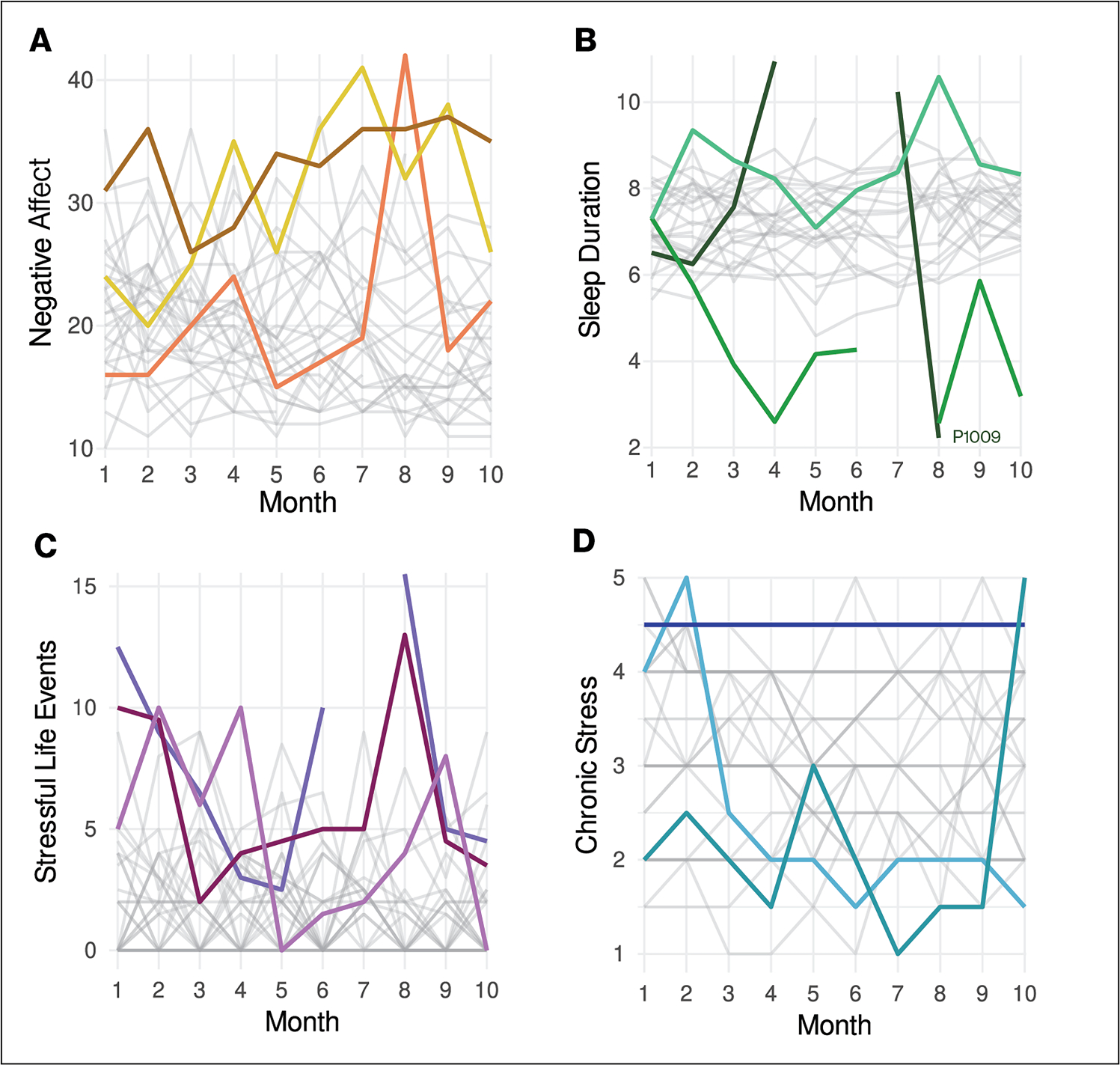
Variation in predictor variables over time. A value for each measurement, for each participant across all 10 months are shown, with separate lines for each participant. Specific participants are highlighted to illustrate examples with high variability or extreme mean values. Discontinuous lines reflect missing data.

The PANAS demonstrated good internal consistency in this sample across months; with Chronbach’s *α* = [.78, .92] (IQR = .81 - .90) across months for negative affect. The ICC(1,1) for negative affect was 0.44 and for positive affect was 0.62, indicating that the majority of variance in negative affect and more than one-third of the variance in positive affect was attributable to within-individual variance (Fig. 6).

**Fig. 7.**
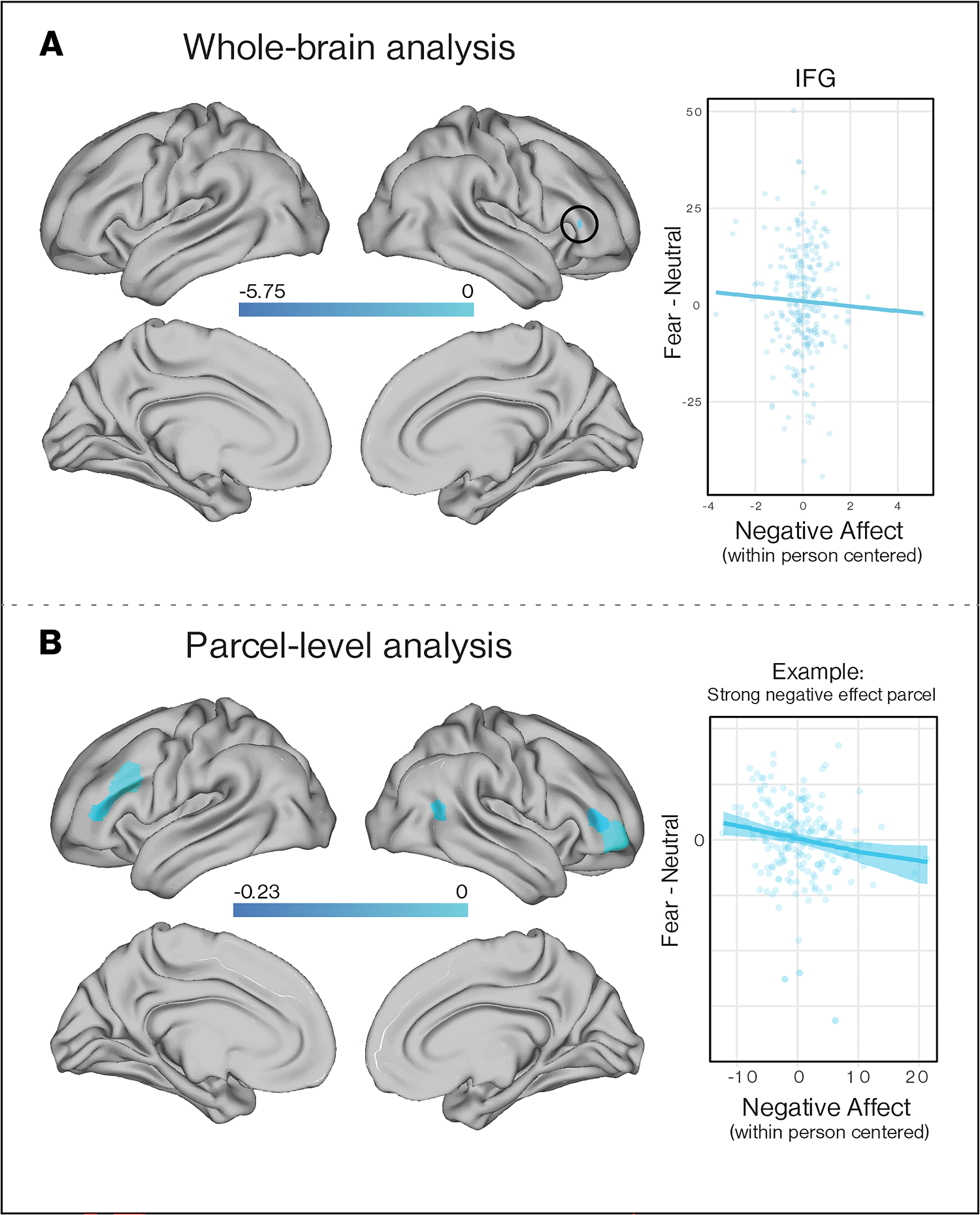
Associations of monthly within-individual fluctuations in negative affect with within-individual variation in neural response to aversive cues. To conduct within-individual analyses, monthly values of each predictor were first centered around each participant’s annual mean; and these annual means, centered at the group mean, were included as a covariate; this approach separates variance at the within-person level from between-person variance. All analyses include month number as a covariate. A: statistical map colors represent t-scores for each effect; values are plotted for voxels within clusters determined to be significant. Scatterplot for the cluster shows mean contrast values on the y axis with the predictor variable on the x axis. The line is the expectation from a model based on mean ROI estimates. B: Parcels with 99.988% credible intervals excluding 0 are displayed using colors representing the effect size for each parcel in terms of standard deviations of the parcel-level outcome for a unit change in the predictor on its original scale. Scatterplots for the example parcels show points for 15 voxels per participant-session with a line for the median of the posterior linear prediction surrounded by the 95% credible interval.

Monthly fluctuations in negative affect were related to within-individual variation in neural responses to aversive stimuli. On months when participants reported higher negative affect than was typical for them, neural responses to aversive cues (fearful > neutral faces) were lower in a small cluster in IFG in whole-brain analysis (Fig. 7A; Table S5). In parcel-level analysis, neural responses to aversive cues were lower in right IFG, MFG, and temporal-parietal-occipital junction (Fig. 7B; Table S6).

#### Sleep.

Sleep duration varied widely over time within-individuals. A majority of the variance in sleep duration occurred within-individuals when examined at the daily level (ICC=0.12) as well as when aggregated across the two-week period prior to each scan (ICC=0.38) (Fig. 6).

**Fig. 8.**
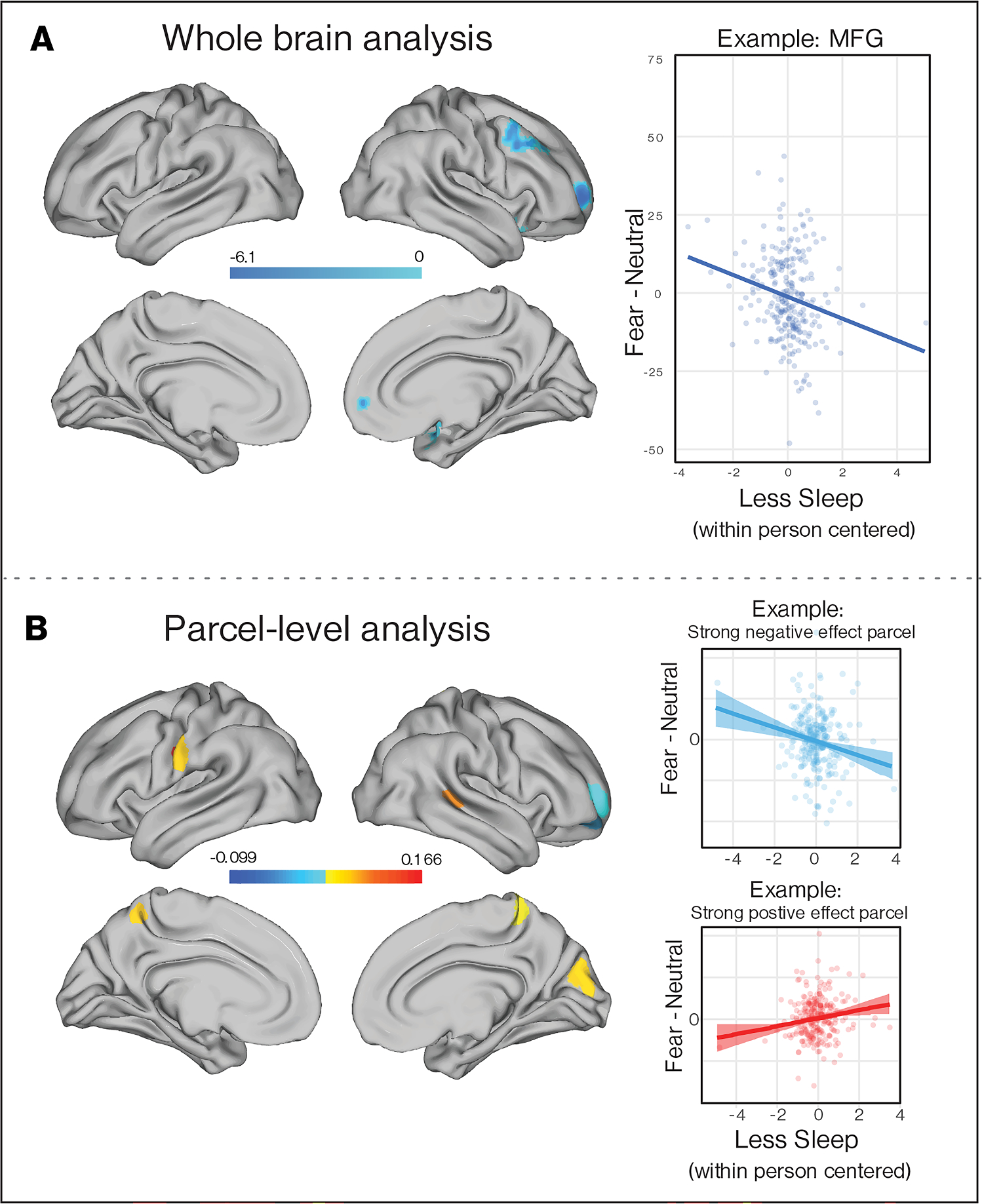
Associations of monthly within-individual fluctuations in sleep duration and within-individual variation in neural response to aversive cues. To conduct within-individual analyses, monthly values of each predictor were first centered around each participant’s annual mean and these annual means, centered at the group mean, were included as a covariate; this approach separates variance at the within-person level from between-person variance. Effect of (shorter) sleep duration on Fear > Neutral contrast. All analyses include month number as a covariate. A: statistical map colors represent t-scores for each effect; values are plotted for voxels within clusters determined to be significant. Scatterplot for the example cluster shows mean contrast values on the y axis with the predictor variable on the x axis. The line is the expectation from a model based on mean ROI estimates. B: Parcels with 99.988% credible intervals excluding 0 are displayed using colors representing the effect size for each parcel in terms of standard deviations of the parcel-level outcome for a unit change in the predictor on its original scale. Scatterplots for the example parcels show points for 15 voxels per participant-session with a line for the median of the posterior linear prediction surrounded by the 95% credible interval.

Within-individual variation in sleep duration was measured objectively using actigraphy in the two weeks preceding the scan was also related to within-individual variability in neural activation. On months characterized by less sleep than usual, participants exhibited lower activation in three prefrontal clusters spanning right (MFG, frontal pole, and frontal orbital cortex in response to aversive stimuli (fearful > neutral faces) in voxel-wise analysis (Fig. 8A, Table S7). In parcel-level analysis, less sleep than usual was similarly associated with reduced activation in right MFG and frontal pole and higher activation in superior temporal sulcus, precuneus, cuneus, and precentral and postcentral gyrus (Fig. 8B, Table S8).

#### Stress.

The ICC for SLEs was 0.25 and 0.70 for chronic stressors, indicating that the majority of variance in exposure to SLEs and about one-third of the variance in chronic stress occurs within-individuals (Fig. 6). The within-individual correlation between SLEs and chronic stressors was r_within_= .25, *p* < .001.

**Fig. 9.**
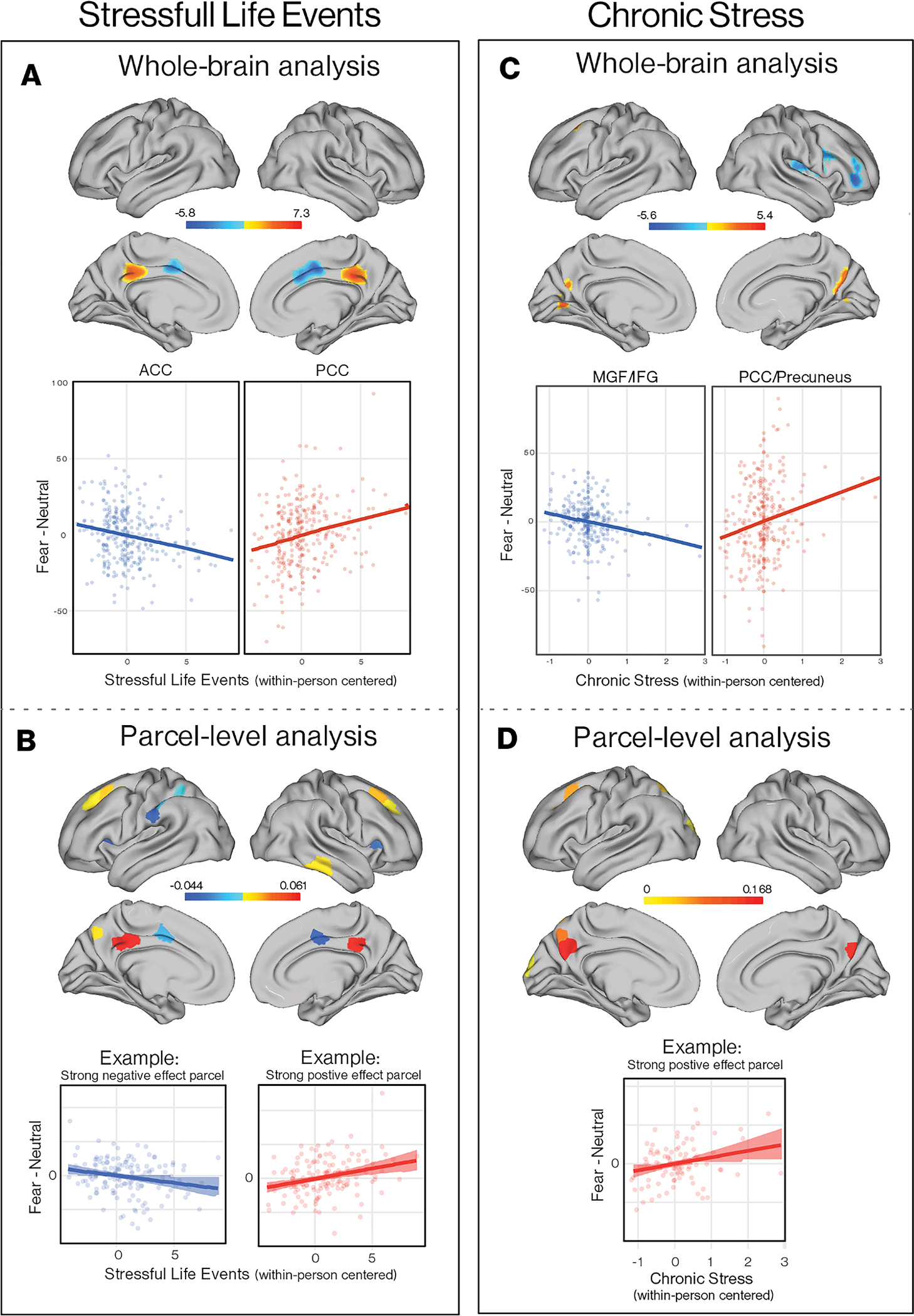
Associations of monthly within-individual fluctuations in stressors and within-individual variation in neural response to aversive cues. To conduct within-individual analyses, monthly values of each predictor were first centered around each participant’s annual mean and these annual means, centered at the group mean, were included as a covariate; this approach separates variance at the within-person level from between-person variance. A, B: Associations of stressful life events with within-individual changes in neural responses in the Fear > Neutral contrast; C, D: Associations of chronic stress with within-individual changes in neural responses in the on Fear > Neutral contrast. All analyses include month number as a covariate. A, C: statistical map colors represent t-scores for each effect; values are plotted for voxels within clusters determined to be significant. Scatterplot for the example cluster shows mean contrast values on the y axis with the predictor variable on the x axis. The line is the expectation from a model based on mean ROI estimates. B, D: Parcels with 99.988% credible intervals excluding 0 are displayed using colors representing the effect size for each parcel in terms of standard deviations of the parcel-level outcome for a unit change in the predictor on its original scale. Scatterplots for the example parcels show points for 15 voxels per participant-session with a line for the median of the posterior linear prediction surrounded by the 95% credible interval.

Monthly fluctuations in both acute SLEs and chronic stressors—assessed using gold-standard interviews—were similarly related to within-individual variation in neural responses to aversive cues. In voxel-wise analysis, adolescents exhibited heightened neural response to aversive stimuli (fearful > neutral faces) in a cluster spanning bilateral PCC and precuneus and reduced neural response in a cluster encompassing bilateral dorsal ACC and dorsomedial PFC on months when they experienced more acute SLEs than was typical for them (Fig. 9A, Table S9). These same clusters were observed in parcel-level analysis, as well as reduced activation in bilateral insula, left superior parietal cortex, and left temporoparietal junction and greater activity in bilateral superior frontal gyrus and lateral inferior temporal cortex (Fig. 9B, Table S10).

For chronic stress, we observed a similar pattern of increased neural response to aversive stimuli (fearful > neutral faces) in one cluster in bilateral precuneus on months when adolescents experienced greater chronic stress than usual. We additionally observed reduced within-individual neural response in a cluster spanning right IFG and MFG and in right putamen on months characterized by higher chronic stress than usual (Fig. 9C, Table S11). The precuneus cluster was also observed in parcel-level analysis, along with elevated activation in left cuneus and superior frontal gyrus (Fig. 9D and Table S12).

#### Amygdala.

Left amygdala was credibly associated with the linear effect of time (Fig. S6). The 95% credible intervals included zero for the association between each other predictor and left and right amygdala activity during emotion processing.

## 4. Discussion

Leveraging a unique sample of adolescents scanned monthly across one year, we investigated the reliability and internal consistency of neural responses during emotion processing over time and within individuals. Although the test-retest reliability of neural responses across time was quite low, internal consistency of BOLD signal across voxels was substantially higher, though this is tempered somewhat by the substantial estimates found in size-matched control regions where no construct-valid variance is expected. Although the ICCs for internal consistency were somewhat lower than standards set for other types of assessments (e.g., interviews, surveys), the estimates obtained here are notable given that they reflect proxy measurement of a complex biological system. Virtually none of the variation in BOLD signal over time reflected stable between-individual differences, whereas nearly half of the variance in cortical parcels reflected internally consistent variance within participants, within sessions, compared to one-third in size-matched control regions. These patterns suggest that instead of reflecting trait-like differences across people, neural responses to aversive cues demonstrate high within-individual variation over time, as well as substantial measurement error when considering the parcel as the unit of measurement. However, while internal consistency was higher in cortical parcels than size-matched control regions, subcortical regions were similar to control regions, suggesting that task response may not be driving signal coherence in subcortical regions. We further demonstrate that this within-individual variability is associated with multiple factors that fluctuate meaningfully over time within individuals. Specifically, within-individual variation in mood, sleep, and exposure to SLEs was associated with dynamic monthly changes in brain activity during emotion processing. These rapid, spatially consistent changes in brain activity suggest that within-individual variation in psychological and physiological states as well as environmental experiences dynamically influence brain activity during adolescence. More broadly, these findings add to growing evidence that precision neuroscience methods have the power to reveal properties of brain function that are not apparent in cross-sectional, between-individual approaches.

The test-retest reliability of BOLD signal in response to aversive cues across the ten neuroimaging sessions was low, indicating poor test-retest reliability of neural responses during emotion processing. This pattern contrasts with two prior reports documenting high reliability in regions of interest for activation during similar emotion processing tasks across two sessions (Gee et al., 2015; Haller et al., 2018). These findings are consistent, however, with a recent meta-analysis and analysis of large cohorts documenting low test-retest reliability of neural response to a range of fMRI tasks, with affective processing tasks exhibiting the lowest reliability (Elliott et al., 2020), and additionally extend the finding of low test-retest reliability to a much shorter time-scale (1 month). These findings have broad implications for affective neuroscience, as these types of emotion processing tasks have been frequently used to study differences in brain function in relation to psychopathology, personality, environmental experiences, and genetics (Canli et al., 2001; Etkin and Wager, 2007; Gray et al., 2005; Groenewold et al., 2013; Hariri et al., 2002; McCrory et al., 2011; Monk et al., 2008; Thomas et al., 2001; Tottenham et al., 2011). Together with the Elliot et al meta-analysis (Elliott et al., 2020), our results suggest that neural responses to affectively-salient cues are poor candidates for research on brain-based biomarkers of stable between-person, i.e., interindividual, variation.

The degree to which this low test-retest reliability extends to neural responses to other types of tasks and metrics of brain activity is an important question for future research. Higher test-retest reliability estimates were observed in the recent meta-analysis for tasks tapping motor and sensory function as well as working memory (Elliott et al., 2020), suggesting that test-retest reliability varies meaningfully as a function of task design and processing domain. Indeed, multivariate patterns of neural response to task demands identified using machine learning may be more stable over time than responses in individual brain areas (Kragel et al., 2021). Functional connectivity of cortical networks assessed using resting-state fMRI may be better suited to studying between-person differences. Although the topography of resting-state cortical networks is highly variable across people (Braga and Buckner, 2017; Gordon et al., 2017), network organization and functional connectivity is highly stable within-individuals over time, with most variance due to stable differences across people (Gratton et al., 2018; Laumann et al., 2017). On the other hand, recent work suggests that the magnitude of brain-behavior associations using resting-state functional connectivity metrics are small and require thousands of individuals to be identified reliably (Marek et al., 2022).

Our findings suggest that poor test-retest reliability of task-evoked BOLD signal likely reflects both high measurement error and high within-individual variability. About half of the variance in cortical parcels and two-thirds in subcortical regions reflects measurement error. The internal consistency of neural response in subcortical regions was only marginally higher than in size-matched control regions in white matter, and ventricles. Virtually all of the remaining variance reflects reliable within-individual variation in the BOLD over time for neural responses during emotional processing. This raises questions about a number of apparently replicable findings that have emerged from between-individual studies of brain activation in these types of emotion processing tasks. For example, elevated neural response in the amygdala and salience network to aversive cues has frequently been observed among people with depression and anxiety disorders (Monk et al., 2008; Swartz et al., 2015b; Thomas et al., 2001) and those who have experienced childhood trauma (McLaughlin et al., 2019). These findings raise the intriguing possibility that rather than reflecting trait-like variability as a function of psychopathology or early-life experiences, these individual differences in neural responses to affective cues may instead reflect state-like factors that vary consistently as a function of psychopathology or exposure to trauma, such as arousal, affect, concentration, sleep, physical activity, or exposure to recent stressors. Future research utilizing dense sampling from the same individuals is needed to explore this possibility.

One solution to dealing with the high measurement error and small effect sizes for brain-behavior associations is to use enormous sample sizes, but another is to utilize the type of densely-sampled longitudinal data of the type we present here (Marek et al., 2022). Within-individual analysis combined with data reduction using parcellations seems to provide good sensitivity in this case. While there is very little reliable between-person variability in neural responses during emotion processing, there is internally consistent systematic variability in BOLD signal fluctuations over time within-individuals. In addition to the methods used here to increase the signal of constructs of interest, this is a potent reminder that there are many other ways to improve signal detection aside from increasing sample size. Still another approach that holds promise in that regard is using individual-level parcellations of network organization given notable individual differences in network topography (Braga and Buckner, 2017; Gordon et al., 2017). Indeed, recent work suggests that brain-behavior associations are larger when individual-specific parcellations are used (Kong et al., 2021, 2019).

It is important to note that there are many possible sources of state variance in the BOLD signal aside from task-related neural activity. Even though covariates can reduce many of these sources, it is not possible to completely eliminate them. For example, BOLD signal may be influenced by heartbeat, respiration, hydration, and motion (Liu, 2016) that are idiosyncratic to a particular person during a particular session, but spatially coherent across voxels in a parcel. Other properties of the MR signal may also contribute to internal consistency of BOLD signal within parcels, for example, aspects of the scanner environment like temperature, humidity, or how many scans were done previously. In the present study, we computed internal consistency for the contrast of fearful relative to neutral faces. The above state-like sources of noise, if they are fairly stable across the duration of the task, are likely somewhat ameliorated when we subtract the signal during neutral blocks from signal during fear blocks. In addition, if spatial coherence in the BOLD signal were primarily responsible for the internal consistency we see within-individuals, we would expect to observe a positive association between smoothness and internal consistency. In contrast, smoothness was unrelated to these estimates. However, the above does not address the broader question of validity. Indeed, the internal consistency of size-matched control regions was non-trivial, indicating that there are sources of spatially coherent but invalid signal. As such, we view the estimates of internal consistency as upper bounds of the true reliability of task-evoked BOLD signal for emotion processing tasks, and certainly as a generous upper-bound on validity.

Importantly, we demonstrate that monthly fluctuations in mood, sleep, and exposure to stress predict variation in neural responses to aversive cues over time within individuals. Notable convergence was observed across these predictors. Adolescents exhibited decreased activation in dorsal and ventral lateral PFC in response to aversive stimuli on months when they reported lower mood, had less sleep, and experienced higher exposure to SLEs than was typical for them; we also observed decreased activation in dorsal ACC on months characterized by less sleep and higher than usual stress. These results demonstrate consistent within-individual reductions in neural response to aversive cues in regions involved in monitoring control-relevant information (e. g., conflict) and implementing control processes (Shenhav et al., 2013). Recent evidence demonstrates shared neural representation of aversive stimuli and cognitive conflict in dorsal ACC, which suggests that aversive stimuli signal control demands similarly to conflict (Vermeylen et al., 2020). Given the absence of a meaningful behavioral response in this task, it is unclear what these neural patterns might reflect (Poldrack, 2011), although it is worth noting that these PFC regions are also recruited in many forms of emotional processing and emotion regulation (Buhle et al., 2014; Etkin et al., 2011). Finally, we observed within-individual increases in activation in PCC and precuneus—key nodes in the default network—in response to aversive stimuli on months characterized by higher levels of SLEs and chronic stress and, to a lesser extent, higher negative mood. PCC activation occurs in response to positive and negative affective stimuli (Maddock et al., 2003), when reflecting on emotional states in oneself and others (Ochsner et al., 2004), and in self-referential processing (Northoff et al., 2006), including auto-biographical memory (Sugiura et al., 2005). It is possible that aversive stimuli are more likely to trigger self-focused thinking, such as rumination, during periods characterized by high levels of stress. Indeed, exposure to SLEs is associated with increased engagement in rumination in longitudinal studies (Michl et al., 2013; Moberly and Watkins, 2008). However, interpretation of these neural patterns is speculative.

To our knowledge, these types of within-individual associations with neural activity have not previously been documented. These patterns add to growing evidence that precision neuroscience, which focuses on repeated sampling of the same individuals, may stimulate progress in characterizing individual variation in brain function. While task-related BOLD signal—at least in response to affective cues—is likely a poor candidate for studying stable individual variation, as some have recently argued (Elliott et al., 2020), it may still be suited to studying changes in brain function within-individual variation over time. Indeed, we show that fluctuations in neural responses over time are associated with relevant processes known to vary over time within-individuals—including mood, sleep, and exposure to SLEs. These findings parallel recent work in psychology demonstrating high within-individual variation in a range of constructs that have historically been studied in between-person designs—including affect, psychopathology symptoms, and physiological markers—and poor correspondence between associations observed in between-participant designs from those derived from within-participant longitudinal studies (Fisher et al., 2018). Our findings suggest that intensive longitudinal designs that probe neural function in the same individuals over time are needed to characterize this within-individual variability, its correlates, and important measurement characteristics like reliability with more focus on the temporal resolution of the underlying constructs.

We focus here on neural responses to affectively-salient stimuli. Affective constructs are known to have high within-individual variability (Fisher et al., 2018). However, even constructs that are more trait-like—such as working memory—show meaningful variation within-individuals over time that are strongly linked to fluctuations, for example, in stress (Sliwinski et al., 2006). Although we cannot extrapolate from the constructs measured here, it is plausible that neural circuits that support a range of cognitive and affective constructs frequently studied in cognitive neuroscience might exhibit meaningful within-individual variability.

The attention to within-individual variation highlights a broader conceptual point about how cognitive neuroscientists design and analyze studies. Observing associations between constructs over time can be an important tool for unraveling causal effects (Collins, 2006; Raudenbush, 2001; Rohrer and Murayama, 2023). Moreover, the degree of variation over time can itself be an important predictor or outcome, as prior research has shown, for example, in sleep (Vidal Bustamante et al., 2020), and emotion perception and experience (Nook et al., 2021; O’Toole et al., 2020). Even in cross-sectional studies, investigators often have repeated measurements, and appropriately interrogating the inherent within-individual variation has been shown to be beneficial for obtaining reliable measurement of selective attention in the Stroop task (Haines et al., 2020). Designing studies to reap the numerous benefits of within-individual variability will fortify the empirical value of our science.

### 4.1. Limitations

There are several aspects of the current study design and set of analyses that should be considered in interpreting these findings. We were not able to address several potential causes of variance in reliability and internal consistency here either because of data limitations or scope. Most importantly, as stated in the introduction, is the fact that we examine reliability and internal consistency, not validity. While reliability is necessary for validity, it is not sufficient.

The particulars of a given parcellation scheme or set of ROIs may impact our estimates. Different parcellations have been shown to affect measurements of functional activity and brain-behavior associations, and the results here would be expected to be influenced by parcellation choice as well (Bryce et al., 2021; Flournoy et al., 2020). We focus on subcortical structures and a widely-used cortical parcellation that divides the cortex into 400 regions based on patterns of task-related activation and functional connectivity at rest (Schaefer et al., 2018). Our estimates, especially for internal consistency, would likely be higher for smaller parcels, and lower as parcel size increases, although registration error might contravene this expectation. Indeed, parcellations based on within-person functional connectivity would likely yield the highest signal reliability (Kong et al., 2019; Xue et al., 2021). The goal here is not to tie our findings to a particular parcellation scheme, but rather to examine internal consistency of BOLD signal to explore to what extent measurement error causes low test-retest reliability across the month-long intervals examined.

We were not able to evaluate test-retest reliability at other timescales, which would be an important next step in corroborating the conclusions drawn from the internal consistency results. Such analyses should first consider the timescale over which test-retest reliability in the particular sensory, cognitive, or affective process in question would be expected. At a minimum, higher test-retest reliability would be expected at extremely short time intervals, with the caveat that well-known habituation effects would need to be accounted for as well. With regard to the behavioral variables examined here, monthly variation has been used in prior studies of within-individual variation in stressful life events and sleep (Nook et al., 2021; Rodman et al., 2021; Vidal Bustamante et al., 2020). Assessing stressful life events at shorter intervals, particularly using the objective interview-based approach utilized here, is not feasible. However, timescales ranging from days to weeks might be more appropriate for capturing meaningful within-individual variation in mood. Additional research is needed to determine the appropriate timescale for assessing within-individual variation in neural function across different modalities.

This data collection was not designed to test the variability of test-retest reliability or internal consistency across multiple fMRI tasks or methods of analysis. Even within the domain of affect processing variation in test-retest reliability would be expected in different kinds of emotion processes, but we were not able to test these ideas here. This analysis is focused on more traditional analysis methods (contrast-based, GLM task fMRI analysis), that remain dominant in the literature, and thus the results here do not generalize to more modern, multivariate methods (Marek et al., 2020, 2019). In general, multivariate methods have been shown to have higher reliability (Kragel et al., 2021), and larger effect sizes (Marek et al., 2020), possibly because they better take advantage of widely distributed neural representations. We would expect that to hold for these data as well, but produce the same dissociation between test-retest reliability and internal consistency. Investigating the reliability of multivariate methods in multiple ways is an important next step in this line of research.

The degree of reliability and internal consistency we document here should be taken as an approximate measure with some caveats. Reliability is not a property of just of a test, but also of the people taking the test; in this case, we report on a particular fMRI task in particular parcels and ROIs in adolescent females. Researchers sampling from other populations, or using different parcels, or a different task, should not derive but the most vague expectations from these results. Moreover, these estimates were generated using two common approaches to estimating reliability, but there is considerably more work to be done in developing measurement models for neural data.

Finally, while we document some internal consistency, we are only able to provide cursory evidence as to the validity of this signal. Supporting the validity of this fMRI task as a measure of differences in emotional processing, we saw associations with mood, sleep, and multiple sources of stress that were expected based on between-person studies (Arnone et al., 2012; Goldstein-Piekarski et al., 2015; Larson and Ham, 1993; Mroczek and Almeida, 2004; Sliwinski et al., 2009; Swartz et al., 2015b, 2015a; Wang et al., 2006). However, we do not have more proximal measures of emotional processing that would allow us to test convergent validity, nor do we have measures that would help us rule out other sources of variance that are distinct from emotional processing, yet would also be expected to correlate with neural responses to this task, such as arousal or attention (Zelkowitz and Cole, 2016). This is, perhaps, the more pressing question for cognitive neuroscience at this point. Validity work is often less of a focus in behavioral tasks than surveys (Clark and Watson, 2019), but is no less important (see Schiavone et al., 2023 for a tool to evaluate multiple validities). We often rely on face validity, but this ignores the possibility that variations in task performance and neural responses may be caused by related but distinct processes. This is an important consideration for a precision-neuroscience approach that seeks to discover biomarkers, and to lead us toward mechanistic explanations of these processes.

### 4.2. Conclusion

Leveraging an intensive longitudinal study with 10 monthly scans per participant, we observe low test-retest reliability of neural responses to aversive cues over time. Using a common approach to assessing internal consistency, we demonstrate that measurement of BOLD signal related to emotion processing is moderately consistent within cortical regions, suggesting that this temporal instability across months may in part reflect high within-individual variation in neural responses to affectively-salient cues in addition to measurement error. Internally consistent within-individual variation accounted for roughly half of the variance in BOLD signal over time in cortical parcels and a third in both subcortical regions and size matched control regions, whereas between-person differences explained virtually none. Within-individual variation in sleep, mood, and stress all contributed to this within-individual variability in neural responses. These findings highlight the importance of evaluating the test-retest reliability of neural responses to other types of fMRI tasks to ensure that fMRI studies are designed in a way that accurately reflects the underlying temporal properties of the construct being measured. Doing so could bring needed nuance to discussions of the validity and reliability of task fMRI data for studying individual variation in brain function.

## Supplementary Material

1

## Data Availability

Code and data repository: https://osf.io/zy92w/.
