# Supplementary material for: A precision neuroscience approach to estimating reliability of neural responses during emotion processing: Implications for task-fMRI
Source: Neuroimage. Author manuscript; Available in PMC 2024 Feb 16. (PMC10872443; doi:10.1016/j.neuroimage.2023.120503)
Supplement: 1 [file NIHMS1958007-supplement-1.pdf]

Supplemental Material for:

A Precision Neuroscience Approach to Mapping Within-Person Variability in Brain Activation During Emotion Processing: Implications for Reliability of Task-Based fMRI

John Flournoy, Ph.D.,[1*] Nessa Bryce, M.A.,[1] Meg J. Dennison, Ph.D.,[2] Alexandra Rodman, Ph.D.,[1] Elizabeth McNeilly, MPP,[3] Lucy Lurie, B.A., Debbie Bitran, B.A.,[4] Azure Reid-Russell, B.A.,[1] Vidal-Bustamante, C.M.,[1] Tara Madhyastha, Ph.D.[5,6]; Katie A. McLaughlin, Ph.D.[1]

[1] Department of Psychology, Harvard University

[2] Australian Childhood Foundation

[3] Department of Psychology, University of Oregon

[4] Department of Psychology and Neuroscience, University of North Carolina at Chapel Hill

[5] RONIN

[6] Department of Psychology, University of Washington

Supplemental text

Sleep and actigraphy methods

Actigraphy devices

For the first few months of the study, participants wore a Microsoft Band 2, but we suspended its use because Microsoft discontinued the model early in the study, in addition to other issues such as short battery life and clunky, uncomfortable wearable design. All participants were then switched to a Fitbit Charge 2. The 16 participants who had started their participation in the study prior to this date were switched to a Fitbit Charge 2 at their next monthly session, and the remaining 14 participants completed their entire study participation using the Fitbit Charge 2. A total of 71.76% of all daily sleep data across participants was collected with the Fitbit device. Reliability analyses were conducted to compare the sleep measurements provided by the two wristbands over 24-hour periods. Four members of our lab wore both devices at the same time (one on each wrist, switching wrists every day) for a period of five days. The results indicated that while sleep duration measurements were very reliable between the two devices (Cronbach's alpha of 0.80), other metrics, such as restless sleep, were substantially less reliable (Cronbach's alpha of 0.51). In order to preserve as much of the sleep data collected in this study while keeping these inter-device differences in mind, the current investigation only includes sleep metrics that were reliably measured across devices. In prior published work on the sleep data from this study, all analyses that used sleep as a predictor in the model were re-run with dummy-coded device source (i.e., Fitbit or MS Band) as an interaction term, and the pattern of associations remained the same for all results (Vidal Bustamante et al., 2020).

Missing Actigraphy Data

Participants were asked to charge their device and to synchronize it with an app-based database every day, and received reminders via text and phone call if they had not synchronized their device for three consecutive days. Participants were also asked to synchronize their data in-person at the monthly visits. Failure to manually sync the data to their phone for a period of seven consecutive days resulted in those data being overwritten and therefore going missing. Aggregating across all participants, 32.47% out of the total 10,106 total study days were missing sleep data (ranging from 1.8% to 88.8% per participant). While it is important to note that six subjects had more than 60% of their maximum possible data missing, the minimum number of available sleep observations per subject was 48 days, which is three to sixteen times more daily data than prior actigraphy-based sleep studies with adolescents to date (Carpenter et al., 2017; Cohodes et al., 2020; Doane and Thurston, 2014; Harbard et al., 2016; Littlewood et al., 2019). To address the possibility that sleep data was not missing completely at random, we used all available data and a maximum likelihood estimator to fit multilevel models. This approach is robust to the data being missing at random, that is, to missingness that is conditional on observed values of the modeled outcome, in this case sleep variables (Matta et al., 2018). It is not possible to determine empirically whether data are missing not at random, as this depends on unobserved values, but it is unlikely that differences in sleep duration or neural function cause participants to remove or turn off their watches.

Model specification and priors for Bayesian models

The following describes the priors used for each Bayesian model extracted directly from model fits.

Test-retest (between-person stability) models

ICC example

```
> test_retest_ex_model$formula
y ~ sess + (1 | id)
> test_retest_ex_model$family

Family: gaussian
Link function: identity

> prior_summary(test_retest_ex_model)
                 prior     class        coef group resp dpar nlpar lb ub       source
                (flat)         b                                              default
                (flat)         b sessmonth02                             (vectorized)
 student_t(3, 0.3, 12.7) Intercept                                            default
   student_t(3, 0, 12.7)        sd                                  0         default
   student_t(3, 0, 12.7)        sd               id                 0    (vectorized)
   student_t(3, 0, 12.7)        sd   Intercept    id                 0    (vectorized)
   student_t(3, 0, 12.7)     sigma                                  0         default
```

Meta-analysis of ICCs example

```
> test_retest_MA_ex_model$formula
est_logit | se(sd_logit, sigma = TRUE) ~ 1
> test_retest_MA_ex_model$family

Family: gaussian
Link function: identity

> prior_summary(test_retest_MA_ex_model)
                 prior     class coef group resp dpar nlpar lb ub  source
 student_t(3, -3.6, 2.5) Intercept                                 default
    student_t(3, 0, 2.5)     sigma                            0    default
```

Internal Consistency (Within-Person Reliability) models

```
> interncons_ex_model$formula
y ~ 1 + sess_fac + (1 | id/sess)
> interncons_ex_model$family

Family: student
Link function: identity

> prior_summary(interncons_ex_model)
              prior     class       coef   group resp dpar nlpar lb ub       source
             (flat)         b                                              default
             (flat)         b sess_fac02                              (vectorized)
             (flat)         b sess_fac03                              (vectorized)
             (flat)         b sess_fac04                              (vectorized)
             (flat)         b sess_fac05                              (vectorized)
```

```
              (flat)         b sess_fac06                              (vectorized)
              (flat)         b sess_fac07                              (vectorized)
              (flat)         b sess_fac08                              (vectorized)
              (flat)         b sess_fac09                              (vectorized)
              (flat)         b sess_fac10                              (vectorized)
 student_t(3, 0, 2.5) Intercept                                             default
       gamma(2, 0.1)        nu                                   1          default
      normal(0, 0.25)       sd                                   0             user
      normal(0, 0.25)       sd                 id                0     (vectorized)
      normal(0, 0.25)       sd  Intercept      id                0     (vectorized)
      normal(0, 0.25)       sd            id:sess                0     (vectorized)
      normal(0, 0.25)       sd  Intercept id:sess                0     (vectorized)
 student_t(3, 0, 2.5)    sigma                                   0          default
```

Smoothness models

Unconstrained model

```
> smooth_rel_s_alt_fit$formula
Estimate | mi(Est.Error) ~ 1 + s(scale_N_voxels, anatomy_fac, bs = "fs") + s(smooth_mean, anatomy_fac, bs = "fs")
phi ~ 1 + (1 | anatomy_fac)
> smooth_rel_s_alt_fit$family

Family: beta
Link function: logit

> prior_summary(smooth_rel_s_alt_fit)
                prior     class                                    coef       group resp dpar nlpar lb ub       source
 student_t(3, 0, 2.5) Intercept                                                                                default
 student_t(3, 0, 2.5) Intercept                                                           phi                  default
 student_t(3, 0, 2.5)        sd                                                           phi        0         default
 student_t(3, 0, 2.5)        sd                                         anatomy_fac       phi        0    (vectorized)
 student_t(3, 0, 2.5)        sd                               Intercept anatomy_fac       phi        0    (vectorized)
   student_t(3, 0, 2)       sds                                                                      0            user
   student_t(3, 0, 2)       sds s(scale_N_voxels, anatomy_fac, bs = "fs")                            0    (vectorized)
   student_t(3, 0, 2)       sds    s(smooth_mean, anatomy_fac, bs = "fs")                            0    (vectorized)
```

Best fitting model

```
> smooth_rel_s_null_null_fit$formula
Estimate | mi(Est.Error) ~ 1 + scale_N_voxels + (1 | anatomy_fac)
phi ~ 1 + (1 | anatomy_fac)
> smooth_rel_s_null_null_fit$family

Family: beta
Link function: logit

> prior_summary(smooth_rel_s_null_null_fit)
                prior     class           coef       group resp dpar nlpar lb ub       source
         normal(0, 1)         b                                                          user
         normal(0, 1)         b scale_N_voxels                                   (vectorized)
 student_t(3, 0, 2.5) Intercept                                                       default
 student_t(3, 0, 2.5) Intercept                                 phi                   default
 student_t(3, 0, 2.5)        sd                                           0           default
 student_t(3, 0, 2.5)        sd                                 phi       0           default
 student_t(3, 0, 2.5)        sd                anatomy_fac                0      (vectorized)
 student_t(3, 0, 2.5)        sd      Intercept anatomy_fac                0      (vectorized)
 student_t(3, 0, 2.5)        sd                anatomy_fac      phi       0      (vectorized)
 student_t(3, 0, 2.5)        sd      Intercept anatomy_fac      phi       0      (vectorized)
```

Power analysis simulation

We have 30 participants, and 292 total observations. To properly estimate statistical power, it is necessary to use simulation methods in which the data generating model reflects expectations of the data to be observed. For detailed guidance on how to perform a power calculation for multi-level models, please see Arend and Schäfer (2019) and Green and MacLeod (2016). We perform the following power analysis using the simr package (Green and MacLeod 2016) in R. We assumed 30 participants (level-2 units) and 292 total observations (level-1 units) with no more than 10 observations nested in each level-2 unit. We assumed an ICC = 0.10. All other parameters are set such that we evaluate standardized regression coefficients between 0.05 and .29. We ran 10,000 total iterations across 13 effect sizes (also see Rodman et al, 2021 for a nearly identical power analysis with small differences in assumptions; see this paper's repository for code).

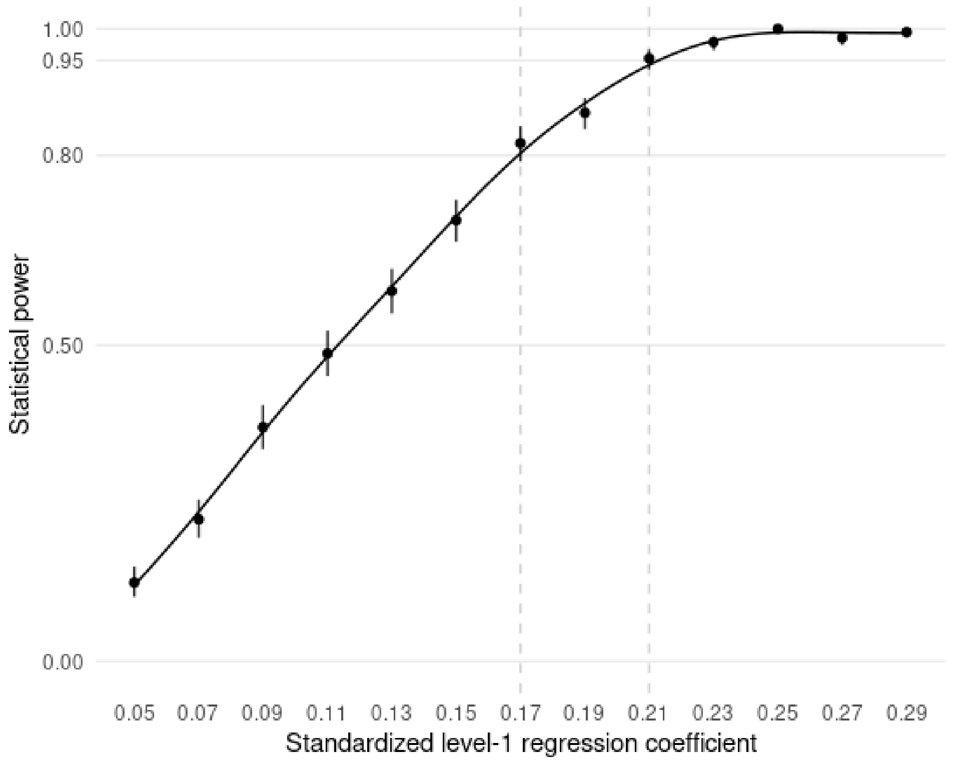

According to this analysis, we would expect power of 80% for effect sizes around B = 0.17 and 95% power for B = .21.

Supplemental Results

Smoothness models

Unconstrained model

```
 Family: beta
  Links: mu = logit; phi = log
Formula: Estimate | mi(Est.Error) ~ 1 + s(scale_N_voxels, anatomy_fac, bs = "fs") + s(smooth_mean, anatomy_fac, bs = "fs")
         phi ~ 1 + (1 | anatomy_fac)
   Data: smoothness_rel_data_full_sum_nomiss (Number of observations: 432)
  Draws: 4 chains, each with iter = 2000; warmup = 1000; thin = 1;
         total post-warmup draws = 4000

Smooth Terms:
                                   Estimate Est.Error l-95% CI u-95% CI Rhat Bulk_ESS Tail_ESS
sds(sscale_N_voxelsanatomy_fac_1) 1.02     0.55     0.30     2.44 1.00        2082     2503
sds(sscale_N_voxelsanatomy_fac_2) 3.52     2.12     0.25     8.51 1.00        1034     1139
sds(sscale_N_voxelsanatomy_fac_3) 10.21    7.17     1.16     28.34 1.00       958      1317
sds(ssmooth_meananatomy_fac_1)    0.66     0.56     0.02     2.12 1.00        1772     2120
sds(ssmooth_meananatomy_fac_2)    1.50     1.42     0.04     5.29 1.00        2199     1948
sds(ssmooth_meananatomy_fac_3)    2.76     3.03     0.10     10.99 1.00       2358     2035

Group-Level Effects:
~anatomy_fac (Number of levels: 3)
                 Estimate Est.Error l-95% CI u-95% CI Rhat Bulk_ESS Tail_ESS
sd(phi_Intercept)        1.47     1.28     0.09     4.74 1.00        1021     1347

Population-Level Effects:
                 Estimate Est.Error l-95% CI u-95% CI Rhat Bulk_ESS Tail_ESS
Intercept        -0.78    0.27     -1.41    -0.37 1.00       923      1610
phi_Intercept    3.32     1.06     0.50     5.12 1.00        1901     1671

Draws were sampled using sampling(NUTS). For each parameter, Bulk_ESS
and Tail_ESS are effective sample size measures, and Rhat is the potential
scale reduction factor on split chains (at convergence, Rhat = 1).
```

Constrained model

```
 Family: beta
  Links: mu = logit; phi = log
Formula: Estimate | mi(Est.Error) ~ 1 + scale_N_voxels + (1 | anatomy_fac)
         phi ~ 1 + (1 | anatomy_fac)
   Data: smoothness_rel_data_full_sum_nomiss (Number of observations: 432)
  Draws: 4 chains, each with iter = 2000; warmup = 1000; thin = 1;
         total post-warmup draws = 4000

Group-Level Effects:
~anatomy_fac (Number of levels: 3)
                 Estimate Est.Error l-95% CI u-95% CI Rhat Bulk_ESS Tail_ESS
sd(Intercept)            0.55     0.55     0.11     2.15 1.00        1072     1314
sd(phi_Intercept)        0.85     0.85     0.06     3.32 1.00        1246     1442

Population-Level Effects:
                 Estimate Est.Error l-95% CI u-95% CI Rhat Bulk_ESS Tail_ESS
Intercept        -0.72    0.42     -1.56    0.22 1.00        1156     953
```

```
phi_Intercept    3.05     0.72     1.14     4.06 1.00        1204     929
scale_N_voxels   -0.15    0.01     -0.17    -0.12 1.00       6562     3071

Draws were sampled using sampling(NUTS). For each parameter, Bulk_ESS
and Tail_ESS are effective sample size measures, and Rhat is the potential
scale reduction factor on split chains (at convergence, Rhat = 1).
```

Model comparison

LOOIC Unconstrained model

```
Computed from 4000 by 432 log-likelihood matrix

         Estimate   SE
elpd_loo    353.8 44.3
p_loo       100.6 41.5
looic      -707.6 88.5
```

LOOIC Constrained model

```
Computed from 4000 by 432 log-likelihood matrix

         Estimate   SE
elpd_loo    424.9 17.7
p_loo         8.8  1.5
looic      -849.7 35.5
```

LOOIC Difference

```
            elpd_diff se_diff
Constrained       0.0     0.0
Unconstrained   -31.2    23.9
```

Supplemental Tables

Table S1. Compensation Schedule
Table S2. Effect of time on contrast of Fear > Neutral
Table S3. Effect of time on contrast of Fear > Neutral, parcellated analysis
Table S4. Between- and within-person correlations among predictor variables
Table S5. Within-person response to aversive cues covaries with within-person negative mood
Table S6. Within-person response to aversive cues covaries with within-person negative mood, parcellated analysis
Table S7. Within-person variation in neural response to aversive cues based on within-person variation in sleep
Table S8. Within-person variation in neural response to aversive cues based on within-person variation in sleep, parcellated analysis
Table S9. Within-person response to aversive cues covaries with within-person stressful life events
Table S10. Within-person response to aversive cues covaries with within-person stressful life events, parcellated analysis
Table S11. Within-person response to aversive cues covaries with within-person chronic stress
Table S12. Within-person response to aversive cues covaries with within-person chronic stress, parcellated analysis

Table S1. Compensation Schedule

Compensation Schedule

Month	Visit Lenth (approx.)	Earnings
1	180 minutes	$ 50.00
2	90 minutes	$ 30.00
3	90 minutes	$ 40.00
4	90 minutes	$ 50.00
5	90 minutes	$ 60.00
6	90 minutes	$ 70.00
7	90 minutes	$ 80.00
8	90 minutes	$ 90.00
9	90 minutes	$ 100.00
10	90 minutes	$ 110.00
11	90 minutes	$ 125.00
12	180 minutes	$ 100.00
	**Total**	$ 905.00

Table S2. Effect of time on contrast of Fear > Neutral

N Voxels	Peak x	Peak y	Peak z	Z	Anatomy
9396	48	-42	4	7.03	Lingual Gyrus
877	-54	-52	14	4.86	Angular Gyrus
328	48	-22	-18	3.56	Middle Temporal Gyrus, posterior division
324	-32	0	-28	3.92	Left Amygdala
213	54	24	18	3.83	Inferior Frontal Gyrus, pars triangularis
132	40	-12	-40	4.35	Temporal Fusiform Cortex, posterior division

Table S3. Effect of time on contrast of Fear > Neutral, parcellated analysis

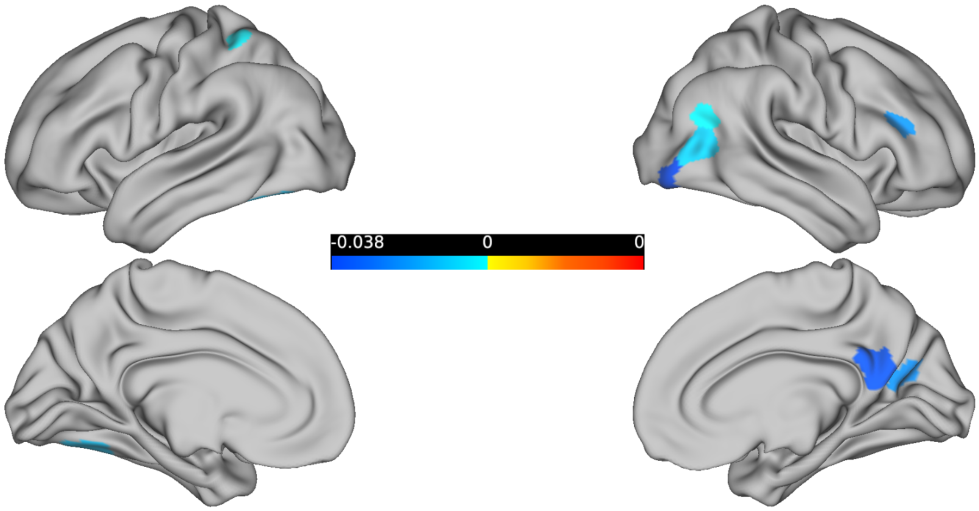

Notes: Estimates are medians of the posterior distributions for the parameter of interest. The 95% credible interval provides one possible range of plausible parameter values. Please see the manuscript for more information about how parcels were selected.

The data for each parcel was scaled by its standard deviation (across all voxels, participants, and waves), which means that the estimates can be interpreted as the expected change in terms of standard deviations of the BOLD contrast for each unit increase in the variable of interest.

**Hemisphere**	**Parcel Label**	**Estimate [95% CI]**	**Location**
Left	Amygdala	-0.025 [-0.044 -0.006]	-
Left	Vis 3	-0.028 [-0.046, -0.009]	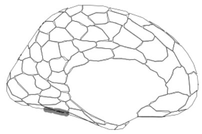
Left	SomMot 27	-0.025 [-0.041, -0.009]	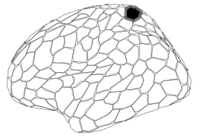
Left	Cont Par 1	-0.024 [-0.042, -0.006]	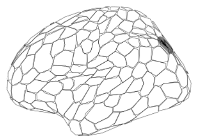

Hemisphere	Parcel Label	Estimate [95% CI]	Location
Right	Vis 10	-0.038 [-0.060, -0.015]	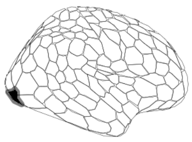
Right	Vis 16	-0.027 [-0.048, -0.006]	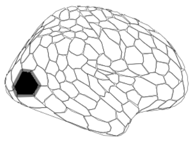
Right	Vis 24	-0.032 [-0.057, -0.007]	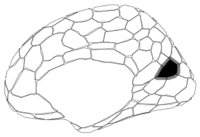
Right	Cont PFCl 6	-0.031 [-0.055, -0.007]	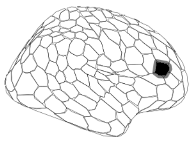
Right	Default Par 2	-0.025 [-0.044, -0.006]	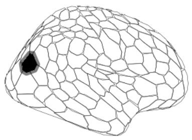
Right	Default pCunPCC 4	-0.037 [-0.058, -0.016]	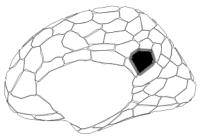

Table S4. Between- and within-person correlations among predictor variables

Between-person

	lower	ϱ	upper
Chronic - Episodic	0.22	0.540.75	
Chronic - PANAS Neg	0.00	0.360.64	
Chronic - Sleep	-0.50	-0.160.21	
Episodic - PANAS Neg	0.02	0.380.65	
Episodic - Sleep	-0.64	-0.360.00	
PANAS Neg - Sleep	-0.51	-0.180.19	

Within-person

	lower	ϱ	upper
Chronic - Episodic	0.14	0.250.35	
Chronic - PANAS Neg	0.09	0.200.31	
Chronic - Sleep	-0.13	-0.010.11	
Episodic - PANAS Neg	0.07	0.180.29	
Episodic - Sleep	-0.17	-0.050.07	
PANAS Neg - Sleep	-0.23	-0.110.01	

Spearman’s Rho with lower and upper 95% confidence intervals.

Table S5. Within-person response to aversive cues covaries with within-person negative mood

N Voxels	Peak x	Peak y	Peak z	Z	Anatomy
94	56	38	8	-5.75	Frontal Pole, IFG pars triangularis

Table S6. Within-person response to aversive cues covaries with within-person negative mood, parcellated analysis

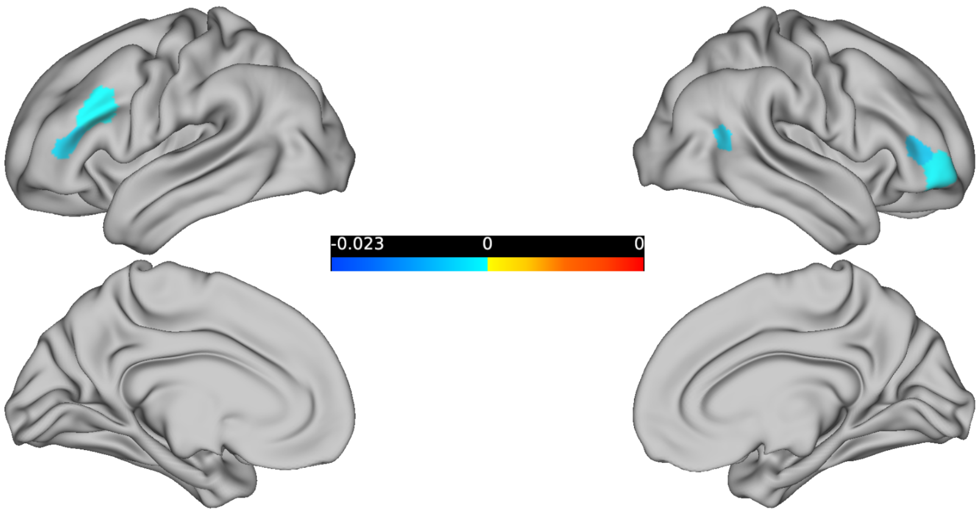

Notes: Estimates are medians of the posterior distributions for the parameter of interest. The 95% credible interval provides one possible range of plausible parameter values. Please see the manuscript for more information about how parcels were selected.

The data for each parcel was scaled by its standard deviation (across all voxels, participants, and waves), which means that the estimates can be interpreted as the expected change in terms of standard deviations of the BOLD contrast for each unit increase in the variable of interest.

Hemisphere	Parcel Label	Estimate [99.988% CI]	Location
Left	DorsAttn Post 5	-0.023 [-0.039, -0.007]	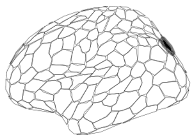
Left	Cont PFCl 2	-0.015 [-0.028, -0.003]	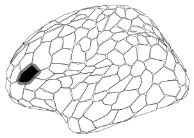
Left	Cont PFCl 6	-0.014 [-0.025, -0.003]	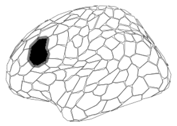

Hemisphere	Parcel Label	Estimate [99.988% CI]	Location
Right	DorsAttn Post 3	-0.015 [-0.029, -0.002]	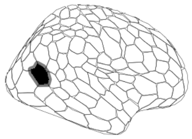
Right	Cont PFCl 2	-0.014 [-0.025, -0.003]	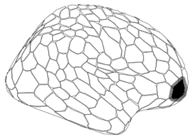
Right	Cont PFCl 4	-0.016 [-0.030, -0.003]	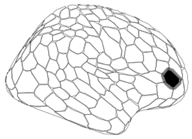

Table S7. Within-person variation in neural response to aversive cues based on within-person variation in sleep

N Voxels	Peak x	Peak y	Peak z	Z	Anatomy
533	24	10	-22	6.1	Frontal Orbital Cortex, Temporal Pole, Insular Cortex
416	34	40	38	4.35	Middle Frontal Gyrus
278	26	60	0	5.93	Frontal Pole

Table S8. Within-person variation in neural response to aversive cues based on within-person variation in sleep, parcellated analysis

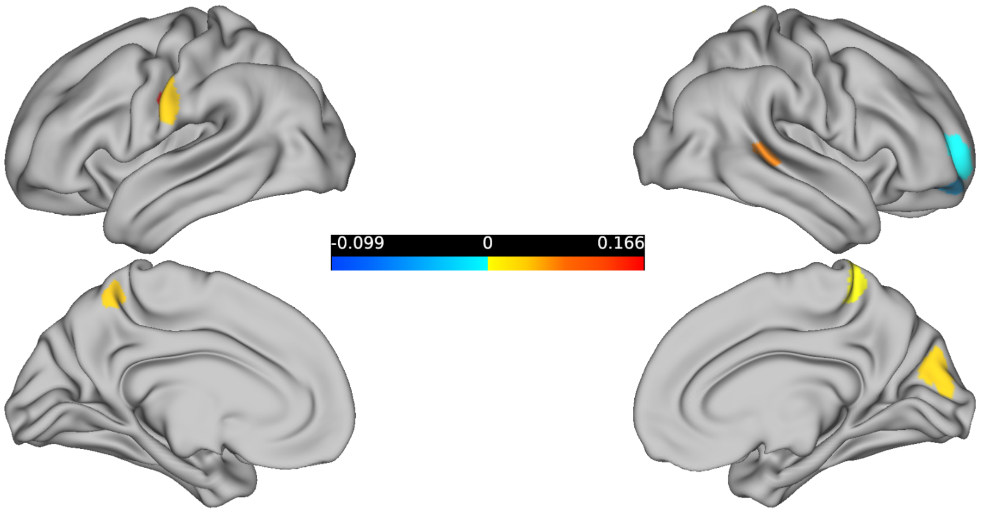

Notes: Estimates are medians of the posterior distributions for the parameter of interest. The 95% credible interval provides one possible range of plausible parameter values. Please see the manuscript for more information about how parcels were selected.

The data for each parcel was scaled by its standard deviation (across all voxels, participants, and waves), which means that the estimates can be interpreted as the expected change in terms of standard deviations of the BOLD contrast for each unit increase in the variable of interest.

**Hemisphere**	**Parcel Label**	**Estimate [99.988% CI]**	**Location**
Left	SomMot 13	-0.094 [-0.165, -0.022]	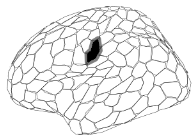
Left	SomMot 14	-0.166 [-0.255, -0.075]	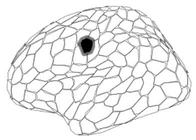
Left	SalVentAttn Med 6	-0.095 [-0.167, -0.022]	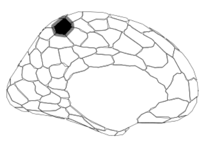

Hemisphere	Parcel Label	Estimate [99.988% CI]	Location
Right	Vis 25	-0.092 [-0.171, -0.016]	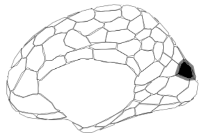
Right	SomMot 32	-0.071 [-0.132, -0.013]	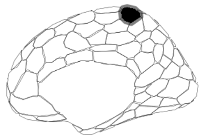
Right	Cont PFCl 1	0.081 [0.017, 0.147]	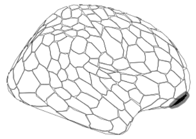
Right	Cont PFCl 3	0.070 [0.011, 0.130]	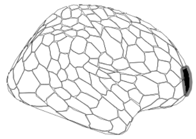
Right	Default Temp 8	-0.113 [-0.192, -0.033]	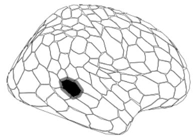
Right	Default PFCv 2	0.099 [0.031, 0.167]	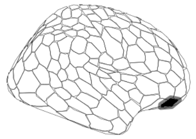

Table S9. Within-person response to aversive cues covaries with within-person stressful life events

N Voxels	Peak x	Peak y	Peak z	Z	Anatomy
943	8.0	-38.0	32.0	7.32	Posterior Cingulate
781	8.0	2.0	44.0	-5.76	Anterior Cingulate

Table S10. Within-person response to aversive cues covaries with within-person stressful life events, parcellated analysis

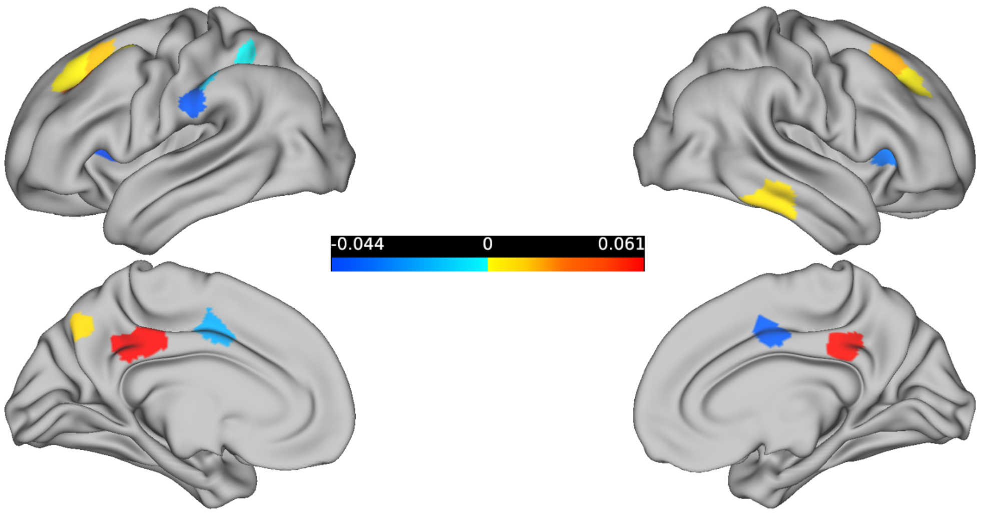

Notes: Estimates are medians of the posterior distributions for the parameter of interest. The 95% credible interval provides one possible range of plausible parameter values. Please see the manuscript for more information about how parcels were selected.

The data for each parcel was scaled by its standard deviation (across all voxels, participants, and waves), which means that the estimates can be interpreted as the expected change in terms of standard deviations of the BOLD contrast for each unit increase in the variable of interest.

Hemisphere	Parcel Label	Estimate [99.988% CI]	Location
Left	DorsAttn Post 6	-0.032 [-0.058, -0.006]	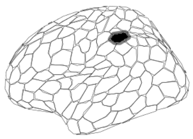
Left	DorsAttn Post 12	-0.029 [-0.049, -0.008]	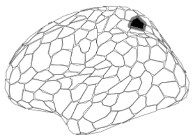
Left	SalVentAttn ParOper 3	-0.042 [-0.069, -0.016]	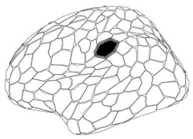

Hemisphere	Parcel Label	Estimate [99.988% CI]	Location
Left	SalVentAttn FrOperIns 5	-0.044 [-0.072, -0.015]	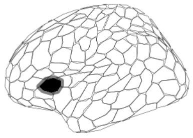
Left	SalVentAttn Med 2	-0.035 [-0.061, -0.008]	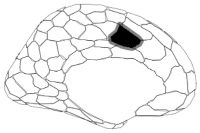
Left	Default PFC 16	0.047 [0.022, 0.073]	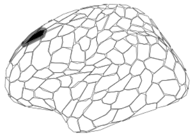
Left	Default PFC 19	0.031 [0.010, 0.052]	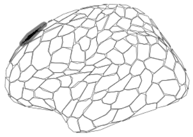
Left	Default PFC 21	0.029 [0.007, 0.052]	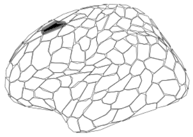
Left	Default PFC 22	0.038 [0.015, 0.062]	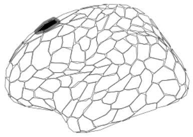
Left	Default pCunPCC 7	0.061 [0.030, 0.091]	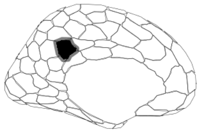
Left	Default pCunPCC 8	0.060 [0.030, 0.090]	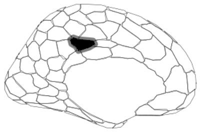

Hemisphere	Parcel Label	Estimate [99.988% CI]	Location
Left	Default pCunPCC 10	0.036 [0.008, 0.062]	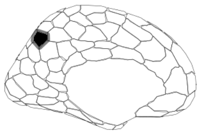
Right	SalVentAttn FrOperIns 5	-0.039 [-0.070, -0.010]	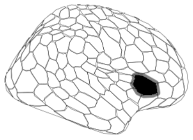
Right	SalVentAttn Med 2	-0.042 [-0.068, -0.016]	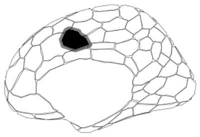
Right	Cont Temp 1	0.034 [0.010, 0.059]	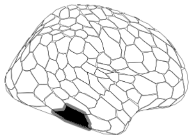
Right	Default PFCdPFCm 10	0.031 [0.007, 0.056]	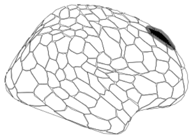
Right	Default PFCdPFCm 12	0.040 [0.017, 0.063]	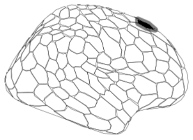
Right	Default pCunPCC 6	0.060 [0.029, 0.091]	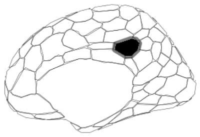

Table S11. Within-person response to aversive cues covaries with within-person chronic stress

N Voxels	Peak x	Peak y	Peak z	Z	Anatomy
1224	30.0	-16.0	10.0	-5.21	R Putamen
660	2.0	-92.0	-18.0	5.39	Lingual Gyrus (midline)
243	44.0	42.0	0.0	-5.59	R Inferior Frontal Gyrus (pars Triangularis)
84	-32.0	16.0	46.0	4.37	L Middle Frontal Gyrus

Table S12. Within-person response to aversive cues covaries with within-person chronic stress, parcellated analysis

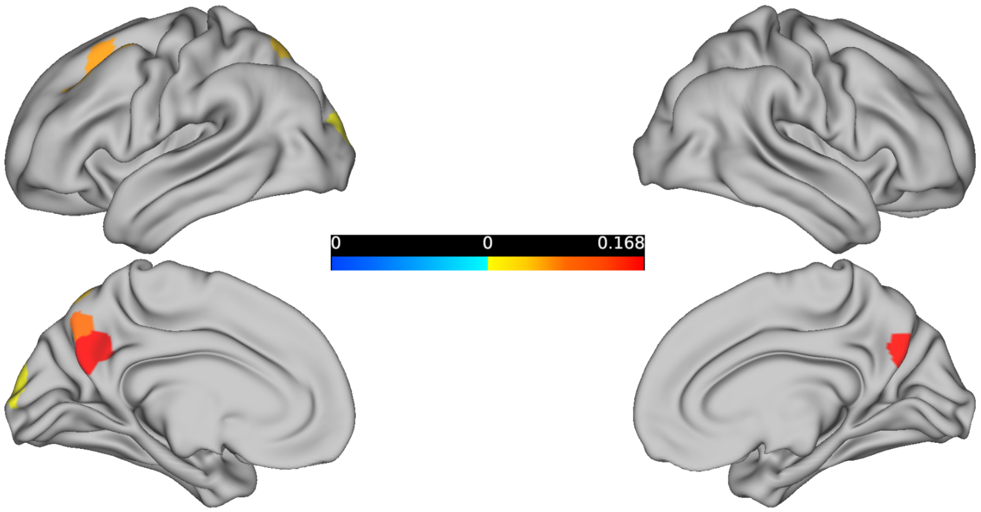

Notes: Estimates are medians of the posterior distributions for the parameter of interest. The 95% credible interval provides one possible range of plausible parameter values. Please see the manuscript for more information about how parcels were selected.

The data for each parcel was scaled by its standard deviation (across all voxels, participants, and waves), which means that the estimates can be interpreted as the expected change in terms of standard deviations of the BOLD contrast for each unit increase in the variable of interest.

Hemisphere	Parcel Label	Estimate [99.988% CI]	Location
Left	Vis 24	0.124 [0.031, 0.220]	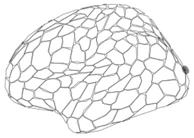
Left	DorsAttn Post 13	0.135 [0.025, 0.250]	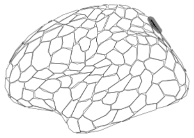
Left	Default PFC 16	0.137 [0.028, 0.248]	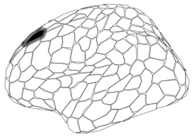

Hemisphere	Parcel Label	Estimate [99.988% CI]	Location
Left	Default PFC 21	0.139 [0.040, 0.235]	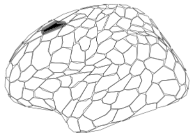
Left	Default PFC 22	0.141 [0.038, 0.247]	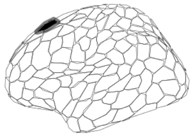
Left	Default pCunPCC 6	0.168 [0.032, 0.306]	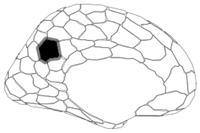
Left	Default pCunPCC 10	0.148 [0.032, 0.265]	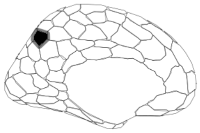
Right	Default pCunPCC 5	0.167 [0.028, 0.308]	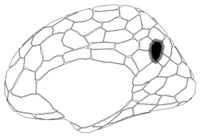

Supplemental Figures

Figure S1. fMRI activation to aversive stimuli in emotion processing task
Figure S2. Temporal stability across all 10 months
Figure S3. Instrument Reliability
Figure S4. Neuropointillist
Figure S5. Zero-order association of size, smoothness and within-person reliability.
Figure S6. Effects on amygdala response to Fear - Neutral

Figure S1. fMRI activation to aversive stimuli in emotion processing task

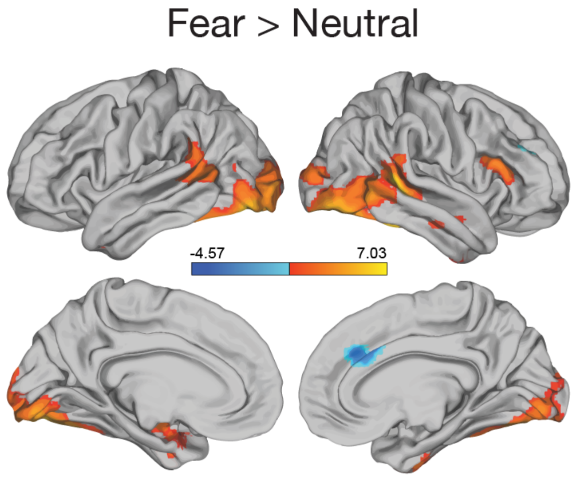

Figure S1. fMRI activation to aversive stimuli in emotion processing task. Statistical map represents t-scores for the average effect of viewing Fear > Neutral faces conditional on study month centered at month 1; values are plotted for voxels within clusters determined to be significant (see Methods for details).

Figure S2. Temporal stability across all 10 months

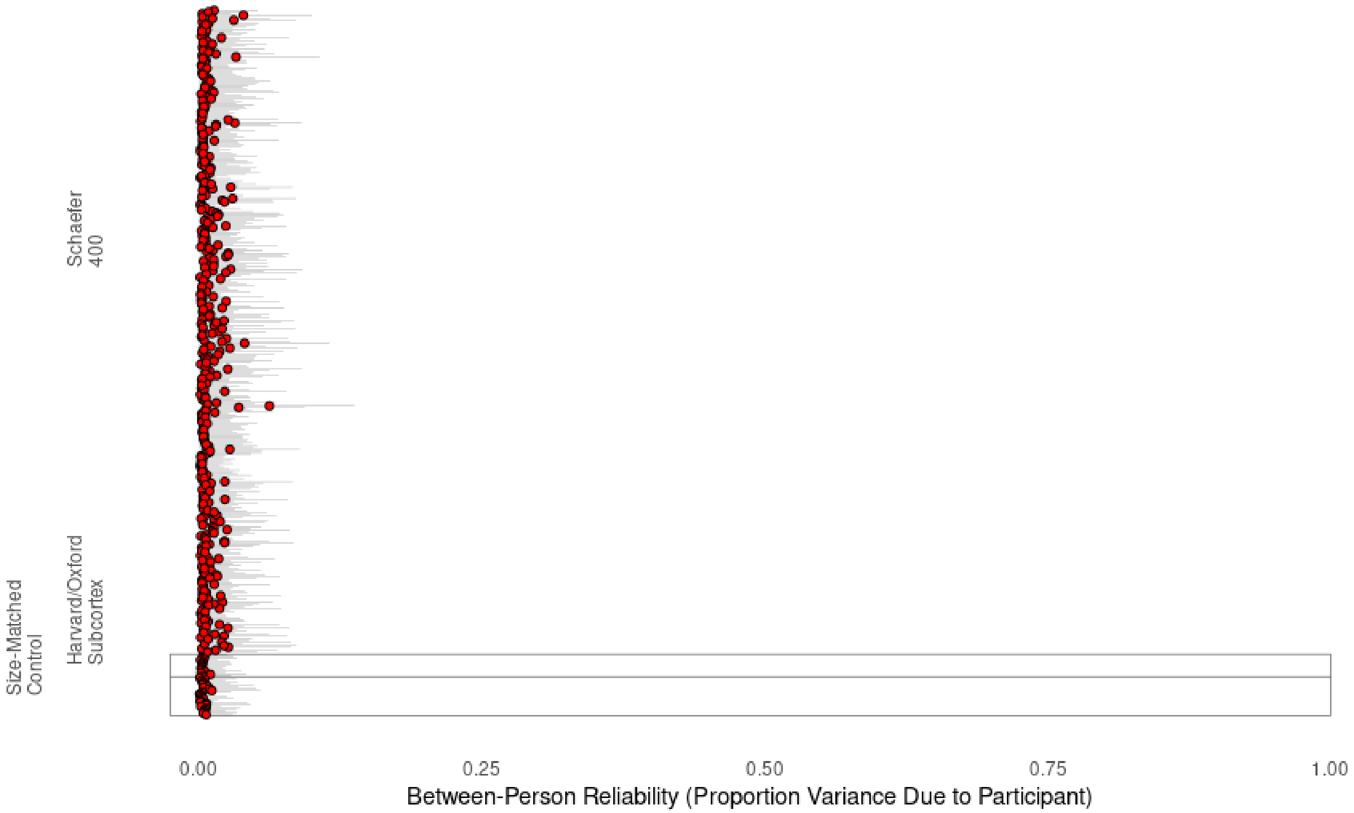

Figure S2. Temporal stability across all 10 months. ICC values and 95% credible intervals of temporal stability averaged across all adjacent pairs of months for each of 400 cortical parcels and 14 sub-cortical regions. Compare to Figure 3A.

Figure S3: Instrument Reliability

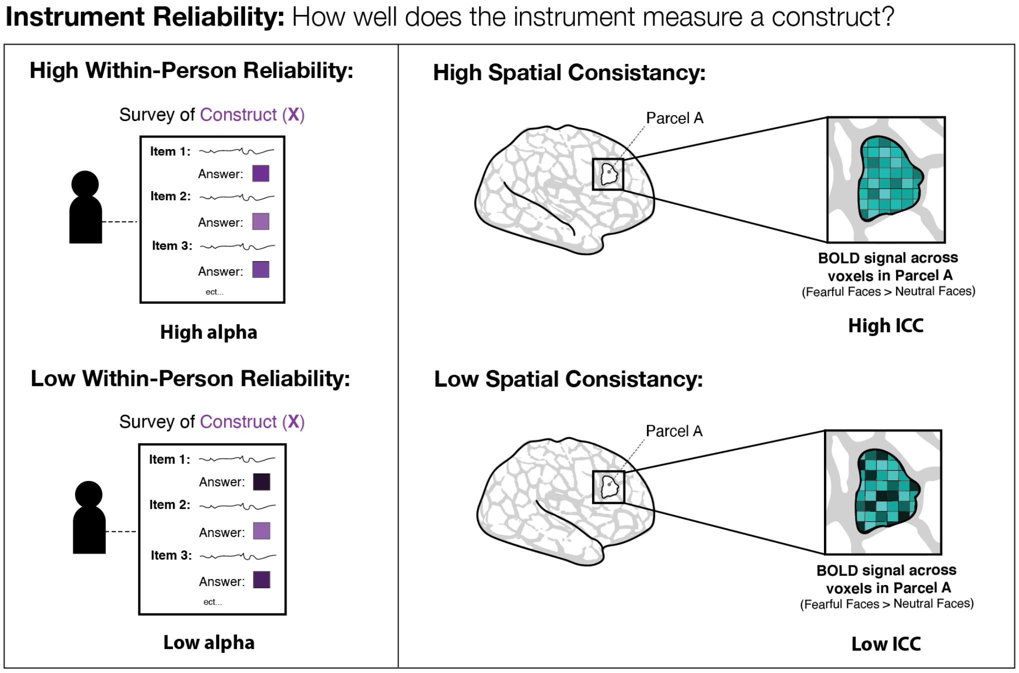

Figure S4. Instrument reliability (internal consistency). The drawing on the left represents how internal consistency is conceptualized as degree of similarity between items on a scale; on the right we show how this can be applied to consistency of activation among voxels within a parcel.

Figure S4: Neuropointillist

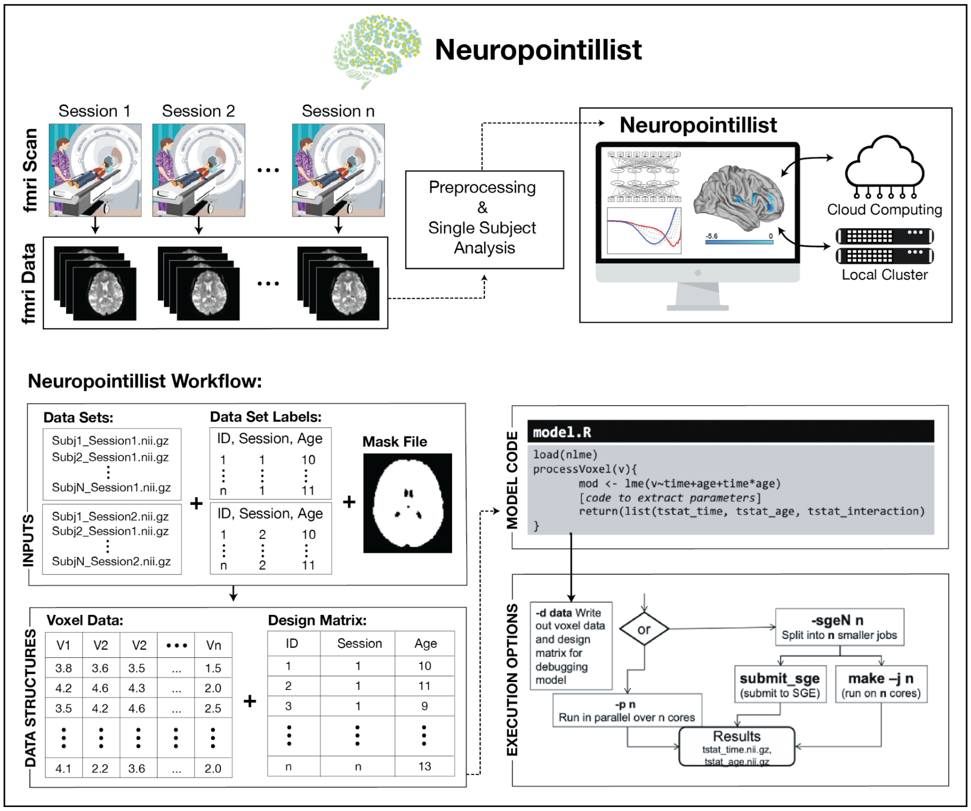

Figure S5. Neuropointillist. Schematic overview of the fMRI analysis software used for this project that allows an arbitrary statistical model, specified in R, to be applied to every voxel in an fMRI data-set.

Figure S5. Zero-order association of size, smoothness and within-person reliability.

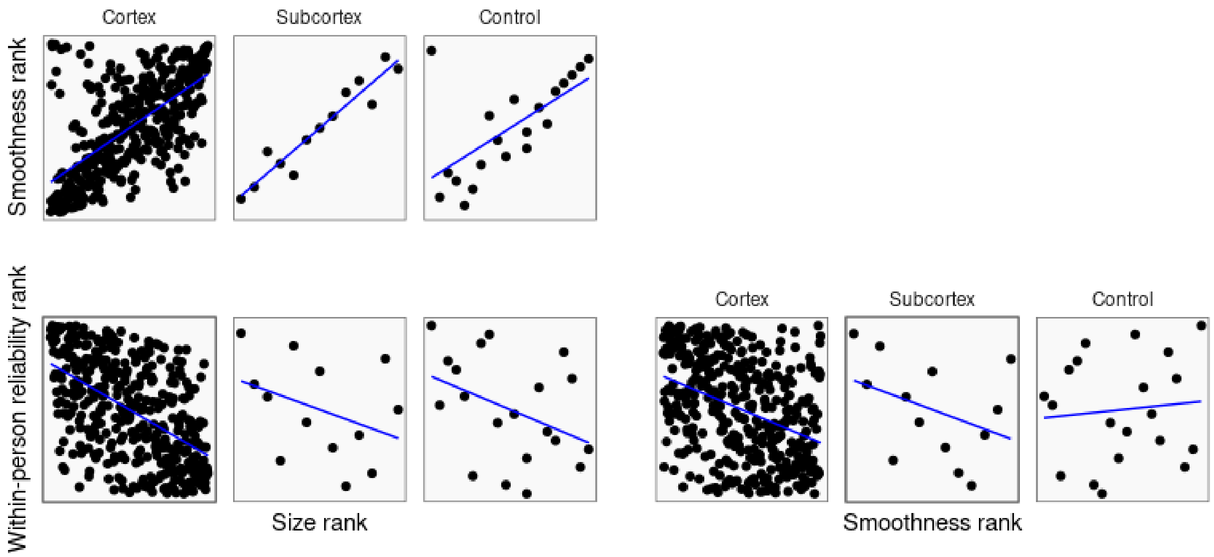

Figure S6. Effects on amygdala response to Fear - Neutral

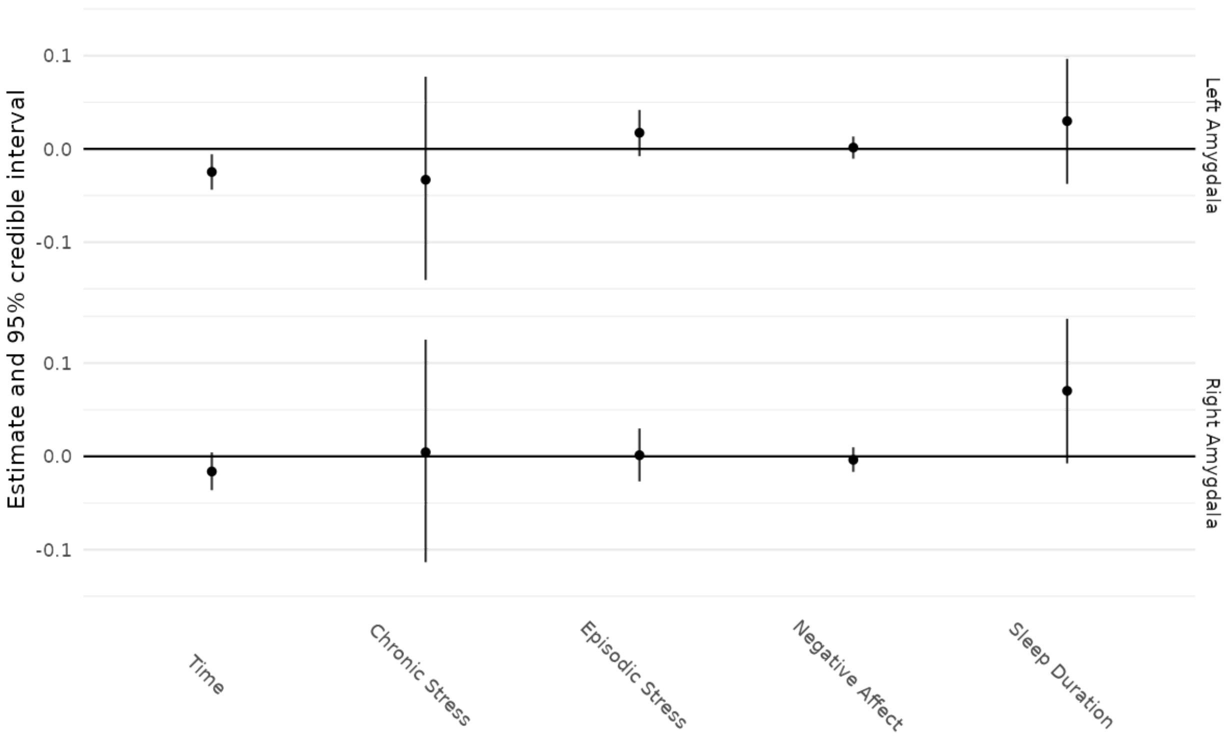

All predictor variables are within-person centered except for time which is the linear effect of session.

Supplemental References

1. Arend, M.G., Schäfer, T., 2019. Statistical power in two-level models: A tutorial based on Monte Carlo simulation. Psychological Methods 24, 1–19. https://doi.org/10.1037/met0000195
2. C. M. Vidal Bustamante et al., Within-person fluctuations in stressful life events, sleep, and anxiety and depression symptoms during adolescence: a multiwave prospective study. Journal of Child Psychology and Psychiatry 61, 1116-1125 (2020).
3. J. S. Carpenter et al., Sleep-wake profiles and circadian rhythms of core temperature and melatonin in young people with affective disorders. (2017).
4. E. M. Cohodes, A. Abusch, P. Odriozola, D. G. Gee, Novel insights from actigraphy: Anxiety is associated with sleep quantity but not quality during childhood. Clinical Child Psychology and Psychiatry 25, 189-199 (2019).
5. L. D. Doane, E. C. Thurston, Associations among sleep, daily experiences, and loneliness in adolescence: Evidence of moderating and bidirectional pathways. Journal of Adolescence 37, 145-154 (2014).
6. Green, P., MacLeod, C.J., 2016. SIMR: an R package for power analysis of generalized linear mixed models by simulation. Methods in Ecology and Evolution 7, 493–498. https://doi.org/10.1111/2041-210X.12504
7. E. Harbard, N. B. Allen, J. Trinder, B. Bei, What's keeping teenagers up? Prebedtime behaviors and actigraphy-assessed sleep over school and vacation. Journal of Adolescent Health 36, 426-432 (2016).
8. D. L. Littlewood et al., Short sleep duration and poor sleep quality predict next-day suicidal ideation: An ecological momentary assessment study. Psychological Medicine 49, 403-411 (2019).
9. T. H. Matta, J. C. Flournoy, M. L. Byrne, Making an unknown unknown a known unknown: Missing data in longitudinal neuroimaging studies. Developmental Cognitive Neuroscience 33, 83-98 (2018).
10. Rodman, A.M., Vidal Bustamante, C.M., Dennison, M.J., Flournoy, J.C., Coppersmith, D.D.L., Nook, E.C., Worthington, S., Mair, P., McLaughlin, K.A., 2021. A Year in the Social Life of a Teenager: Within-Persons Fluctuations in Stress, Phone Communication, and Anxiety and Depression. Clin. Psychol. Sci. 2167702621991804. https://doi.org/10.1177/2167702621991804
11. A. M. Winkler, G. R. Ridgway, M. A. Webster, S. M. Smith, T. E. Nichols, Permutation inference for the general linear model. NeuroImage 92, 381-397 (2014).
12. A. M. Winkler, M. A. Webster, D. Widaurre, T. E. Nichols, S. M. Smith, Multi-level block permutation. NeuroImage 123, 253-268 (2015).
13. D. Freedman, D. Lane, A nonstochastic interpretation of reported significance levels. Journal of Business & Economic Statistics 1, 292-298 (1983).
